# Musculoskeletal study of cebocephalic and cyclopic lamb heads illuminates links between normal and abnormal development, evolution and human pathologies

**DOI:** 10.1038/s41598-018-37735-9

**Published:** 2019-01-30

**Authors:** Rui Diogo, Daria Razmadze, Natalia Siomava, Nora Douglas, Jose S. M. Fuentes, Andre Duerinckx

**Affiliations:** 1 Department of Anatomy, Howard University College of Medicine, Washington, USA; 2 Borissiak Paleontological Institute of Russian Academy of Sciences, Moscow, Russia; 3 Kumangui research group, University Francisco Jose de Caldas, Bogota, Colombia; 4 Department of Radiology, Howard University College of Medicine, Washington, USA

## Abstract

This paper is part of the emerging field of Evolutionary Developmental Pathology, dedicated to study the links between normal and abnormal development, evolution and human pathologies. We analyzed the head musculoskeletal system of several ‘natural mutant’ newborn lambs displaying various degrees of abnormality, from mild defects to *cebocephaly* and to *cyclopia*, and compared them with humans. Interestingly, muscle defects are less marked than osteological ones, and contrarily to the latter they tend to display left-right assymetries. In individuals with cebocephalic and even cyclopic skulls almost all head muscles are normal. The very few exceptions are some extraocular muscles and facial muscles that normally attach to osteological structures that are missing in the abnormal heads: such muscles are instead attached to the ‘nearest topological neighbor’ of the missing osteological structure, a pattern also found in cyclopic humans. These observations support Alberch’s ill-named “logic of monsters” - as a byproduct of strong developmental/topological constraints anatomical patterns tend to repeat themselves, even severe malformations displayed by distantly related taxa. They also support the idea that mammalian facial muscles reverted to an ancestral ‘nearest-neighbor’ muscle-bone type of attachment seen in non-vertebrate animals and in vertebrate limbs, but not in other vertebrate head muscles.

## Introduction

The links between normal and abnormal development, evolution, and pathologies begun to be intensively studied centuries ago by authors such as St. Geoffroy St. Hilaire, but were then somewhat neglected for decades during the 20th century^1^. Fortunately more and more authors are realizing the importance to investigate these links, and such studies are emerging as part of a field that has been named Evolutionary Developmental Pathology (Evo-Devo-Path: see e.g.^1–4^). In particular, it has been recognized that the vast majority of the studies on these links have focused mainly on osteological or superficial features (e.g., absence of a certain bone, shape of head), with almost no information been available about the muscular system of non-human animals with severe malformations.

**Figure 1:**
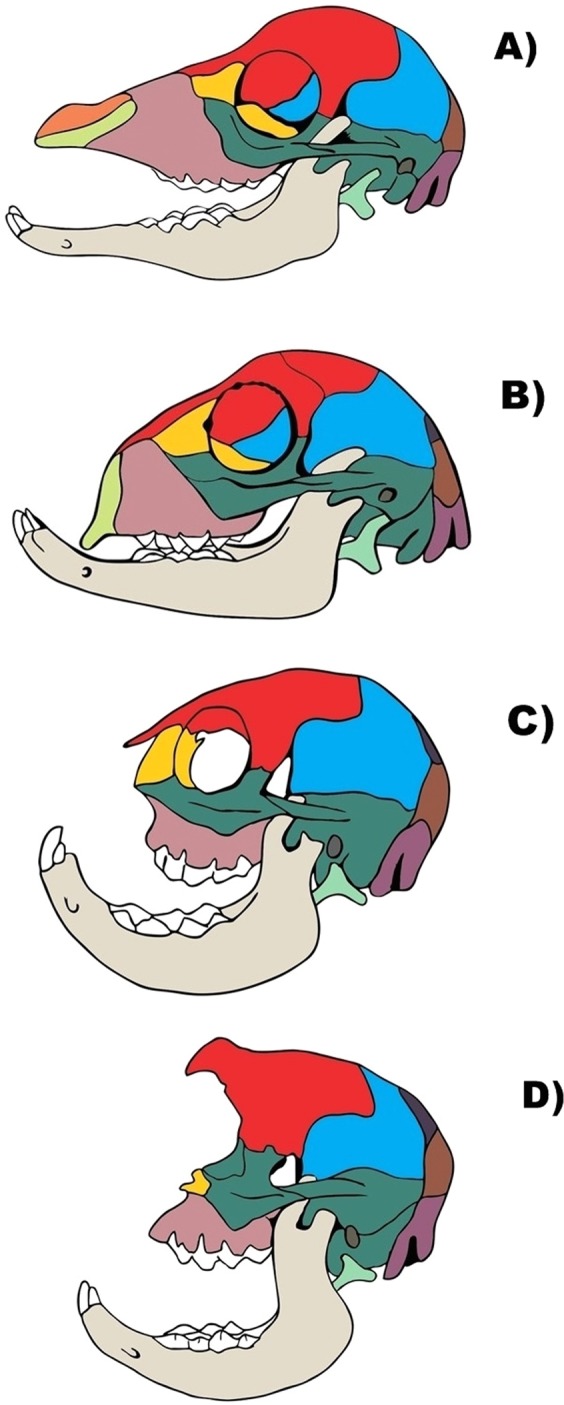
Lateral view of skulls of newborn lambs, showing different osteological stages. (**A**) Stage 1 (*normal* sensu Evans *et al*.^6^). (**B)** Stage 2 (i.e. mildly defective, between *normal* and *cebocephalic* sensu Evans *et al*.^6^). (**C)** Stage 3 (*cebocephalic* sensu Evans *et al*.^6^). (**D)** Stage 4 (*cyclopic* sensu Evans *et al*.^6^). Lighter green: premaxilla; light green: hyoid; dark green, dark brown and blue: temporal, malar and parietal; orange: nasal; light brown: maxilla; yellow: lacrimal; magenta: occipital; red: frontal. For more details see text.

The present paper is part of an ongoing effort to change this status quo. Specifically, we dissected 27 sides (left and right: see SI1 Table 1) of heads of abnormal newborn lambs that were collected during various years by Howard Evans and colleagues to illustrate various degrees of skull (osteological) abnormality, from mild defects (stage 2 sensu the present work, stage 1 being a normal phenotype) to *cebocephalic* (stage 3 sensu the present work) and *cyclopic* (stage 4 sensu the present work) configurations (see^5^ and Fig. 1). Both cebocephaly and cyclopia are considered to be part of the spectrum of *holoprosencenphaly*, which is a term given to a group of forebrain defects characterized by a failure of the prosencephalon to cleave sagitally into cerebral hemispheres, transversely into telencephalon and diecephalon^6^. In cebocephaly - a name that was, interestingly, created by the true grandfather of current evo-devo-path, Geoffroy St. Hilaire - ocular hypotelorism is associated with a ‘monkey-like head’, with a defective small, flattened nose with a single nostril or absent nose and closely set eyes^6^. Cebocephaly, is considered to represent a link between milder forms of holoprosencephaly and cylopia - a condition where a single median orbit contains variably paired ocular structures and where a supraorbital (dorsal) proboscis may or not be present^6^.

Currently, holoprosencephaly is usually classified into three main subtypes based upon its severity: lobar, semilobar, alobar; there is also another subtype, often milder, the middle interhemispheric (MIH) variant^7^. In *alobar holoprosencephaly* there is a total failure of the brain to split into left and right hemispheres: the midline structures of the brain and face are lost, and the lateral ventricles and third ventricle are fused. It some cases there is a single eye (cyclopia) or the eyes are very close to each other (ethmocephaly) or are missing (anophthalmia), or are very small (microphthalmia) with a proboscis. In *semi-lobar holoprosencephaly* the left and right sides of the brain are fused in the frontal and parietal lobes; in some cases there is hypotelorism, anophthalmia, or microphthalmia. In *lobar holoprosencephaly* the left and right ventricles are present but there is a fusion of the cerebral hemispheres in the frontal cortex: phenotypes may include closely spaced eyes, bilateral cleft lip, or a depressed nose. In *middle interhemispheric variant* there is a fusion of the brain, in the middle: phenotypes may include a narrow and depressed nose, or closely spaced eyes.

We complemented our muscle dissections of holoprosencephalic lambs with osteological information obtained from CT scans taken previously to the dissections, and with both gross and morphometric osteological analyses done after the dissections of each muscle (see Materials and Methods). We also compiled the osteological information provided in the literature about other holoprosencenphalic newborn lambs studied by Evans and colleagues, for instance in their influential 1966 paper^5^ (see Fig. 1), which did not include myological analyses. The present paper is therefore the first one including detailed information about the muscles of the holoprosencenphalic newborn lambs collected by those researchers, which are a precious case to study teratogenesis because they concern an example of ‘natural mutants’. In fact, as stressed by those authors more than four decades ago, “the observations of the natural occurrence of (cebocephaly and) cyclopia and related cephalic deformities in sheep are of interest and relevance in so far as they contribute to an understanding of the etiology and epidemiology of corresponding deformities in man”^5:707^. It is actually striking that 1% to 8% of the lambs to which they were referring to - i.e., lambs born from ewes that had been pastured during the breeding season (early August) on certain alpine meadows in southwestern Idaho - displayed such malformations of the head. The high incidence of these anomalies was associated with ingestion around the 14th day of gestation of *Veratrum californicum*, one of the various poisonous range plants of the alpine-type meadows^8–11^. The connection to human medicine is therefore a very direct one here, because plants of the *Veratrum* group have been used for medicinal purposes for centuries and were often used in the second half of the 20th century in late pregnancy, for hypotensive effects: the results with the ground plant or extract were erratic and sometimes fatal for humans^5^. In fact, since the discovery that ingestion of this plant by pregnant sheep induced holoprosencephalic-like craniofacial defects in offspring, numerous investigations have attempted to characterize the mechanisms underlying the induction of teratogenic effects (James *et al*.^12^). For instance, it has been demonstrated that cyclopamine was the primary alkaloid from this plant exerting such teratological effects on developing embryos by selectively blocking Sonic hedgehog signal transduction; since then cyclopamine was used as a probe to understand the biological development of a variety of mammalian, including human, organs, and as a potential therapeutic agent for the treatment of tumors arising from the disruption of the Hedgehog pathway^12^.

In the present paper we therefore provide the first detailed myological description of the holoprosencenphalic newborn lambs collected by Evans and colleagues, and examine if the different degrees of osteological defects reported by them have a one-to-one match with distinctive degrees of muscular defects. Specifically, Evans *et al*. described *four* types of defects in the newborn lambs collected by them based on osteological and external features, which are used in the present paper as the four stages of osteological defects (see Fig. 1) that will be contrasted to the levels of defects found in the musculature^5:708–713^. As noted by them: “gradations of facial defects as seen in sheep can be arranged in various series depending upon the primary structure being considered the most obvious external features such as the eyes, nose, and mouth each have their own range of malformation and occur in different combinations; two basic types of defect can be considered: one is characterized by paired orbits and a reduced olfactory region (cebocephalia), while the other typically has a single median orbit and great reduction or absence of olfactory structures (cyclopia)”. Osteological stage 1 sensu the present work corresponds to their ‘normal newborn lambs’, while stage 2 corresponds to their ‘mildly affected cebocephalic lambs’ (Fig. 1). They describe the latter as lambs having “a shorter muzzle than normal and, thus, a shorter nasal cavity, the eyes appear to be closer together (hypotelorism) because of reduced facial region, the palate and nasopharynx are likewise shortened, the premaxilla is present but smaller, the brain appears normal, and the defects overall are not incompatible with life although the animal may have difficulty in feeding and grow more slowly”. Osteological stage 3 in Fig. 1 corresponds to their “severely affected cebocephalic lambs”, which they define as: “neonatal death, have a short muzzle and a shallow nasal cavity, the cribriform plate is reduced or absent, the orbits are in communication medially, and the nasopharynx fails to develop, resulting in choanal atresia, the premaxilla does not develop in the severely affected animal, the maxilla is greatly reduced, and this results in crowding and deformation of the maxillary teeth”. Lastly, osteological stage 4 (Fig. 1) corresponds to their “cyclopic lambs”. As described by them: “this extreme condition incompatible with life is characterized externally by a single median eye, partially fused eyes, or paired eyes in a median orbit if it bears an external nose at all, has an elongate, tubular proboscis which usually contains cartilage remnants and has been displaced dorsally by the median orbit and is attached above the eye; the skull is much shortened and rounded because of the absence of an ethmoid region, as a result the premaxilla and vomer are absent; the nasal bones are reduced to a plaque at the base of the proboscis or lost”.

Interestingly, Evans *et al*. noted that, osteologically, human cephalic malformations typical of cebocephaly and cyclopia “clearly appear to have their morphologic counterparts in the deformed sheep”. It is therefore crucial to examine if such similarities between lambs and humans are also seen in the muscular system^5:713^. The main aim of the paper is therefore to examine this issue and in particular to thus test Pere Alberch’s ill-named idea that there is a ‘logic of monsters’ by examining if e.g. defective muscles display similar (including homoplasic) configurations in distantly related animals such as humans and sheep as a byproduct of strong developmental/topological constraints, as predicted by Alberch^13^ (for more details, see Discussion). For this purpose we compare our observations of musculoskeletal defects in *cebocephalic* and *cyclopic* newborn lambs with the musculoskeletal defects that are often found in humans with cyclopia, based on our recent dissections and literature reviews of the human phetotypes (compiled in^4^). It is therefore hoped that this paper will further stimulate the development of evo-devo-path in general, and will in particular be useful to comparative anatomists, evolutionary biologists, developmental biologists, evodevoists, veterinarians, zoologists, pathologists and physicians.

## Results

Here we provide just a short summary of the results obtained from our dissections and morphometric analyses, as detailed descriptions of each head muscle dissected by us in each specimen included in the present work are given in SI1 Table 1 and the results of our morphometric analyses are given in SI2.

**Figure 2:**
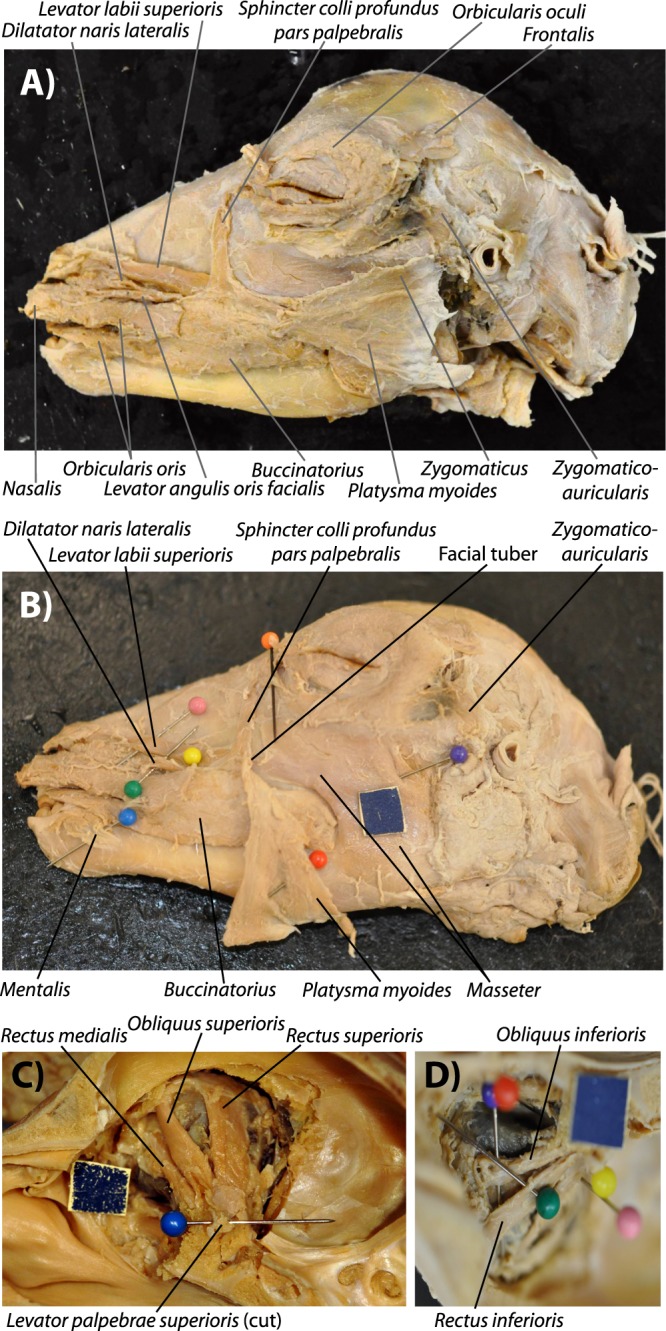
Normal head muscles of newborn lambs, as seen in ‘myological mild stage 2’. The typical configuration of more superficial (**A**) and deep (**B**) facial and masticatory muscles, as well as of extraocular muscles of the left eye (**C**,**D**) of the mildly defective myological stage 2 newborn lambs dissected by us is basically similar to the normal newborn lamb myological phenotype (paper square = 1 cm). That is, basically the face/snout is slightly truncated, but all the head muscles are present and have a mainly normal configuration, as shown here in specimens 12 L (**A**,**C**) and 16 L (**B**,**D**). For more details see text and SI1 Table 1.

**Figure 3:**
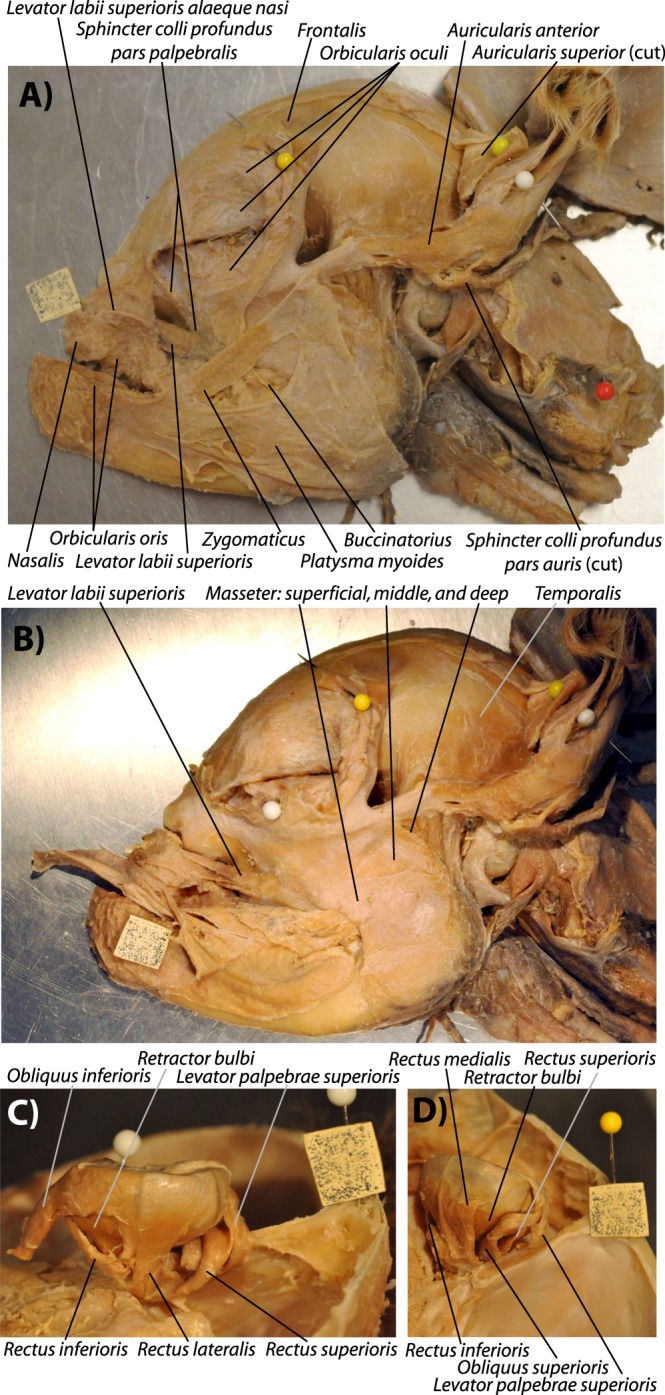
Head muscles of newborn lambs with a ‘myological more defective stage 2’. Typical configuration of more superficial (**A**) and deep (**B**) facial and masticatory muscles, as well as of extraocular muscles of the left eye (**C**,**D**) of more defective myological stage 2 newborn lambs dissected by us, as shown here in specimen 6 (paper square = 1 cm). Basically, in this specimen the truncation of the head is even more marked than in myological mild stage 2, with the facial tuber lying normally below they eye (not markedly anterior to it as is the case in normal newborn lambs), so although all the muscles are present and have their normal attachments, the direction of muscles such as the sphincter colli pars palpebralis are different from normal. This contrasts with stage 3, in which there are 3 small muscles on the snout region (dilatator naris lateralis, levator labii superioris, and levator anguli oris facialis) on the left side and only two on the right side. For more details see text and SI1 Table 1.

The typical configuration of the head muscles of the osteologically less defective (mild stage 2, between *normal* and *cebocephalic* sensu Evans *et al*.^5^, stage 1 being completely normal: see Fig. 1) newborn lambs dissected by us is basically similar to the normal newborn myological lamb phenotype (Fig. 2). That is, the face/snout is slightly truncated (two nostrils and two eyes being present) but all the head muscles are present and have a mainly normal configuration. Within the osteologically abnormal newborn lamb specimens dissected by us the ones displaying such a completely normal muscle configuration were specimens 12, 10 and 16 (Fig. 2). Individuals such as specimen 6 (Fig. 3), as well as the osteologically slightly less defective specimens 2 and 5, display also myological stage 2, in the sense that all muscles are present and basically have their normal attachments, but the face/snout is markedly more truncated than in the previously described specimens, and the facial tuber of the maxillary bone lies much more posterior than it does in normal newborn lambs (see Fig. 2B). It is in fact interesting that despite the marked truncation of the face/snout seen in individuals such as specimen 6, all the muscles basically have the normal attachments, although of course the orientation of some of them is affected by the truncation of the skull. For instance, the origin of muscles such as the levator labii superioris is still from the facial tuber, but because this tuber lies more posterior than in normal newborn lambs, lying actually below the level of the eye and not markedly anterior to it, the direction and length of the muscle is different from that seen in more mild stage 2 newborn lambs and in normal newborn lambs (compare, e.g., Fig. 3 with Fig. 2).

**Figure 4:**
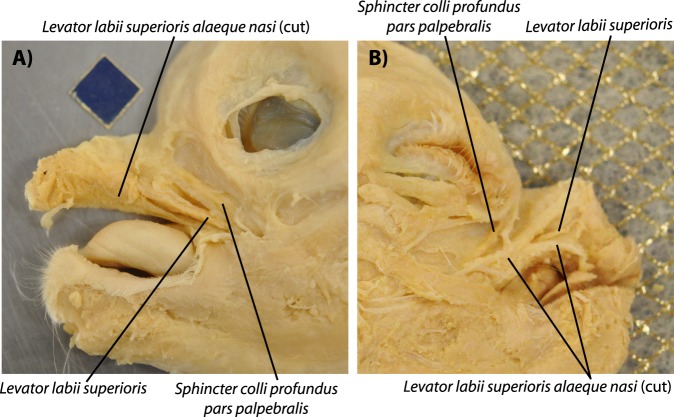
Lateral view of head muscles of a ‘myological stage 3.5’ newborn lamb. Left (**A**) and right (**B**) sides of specimen 122B, showing a condition between myological defective stages 3 and 4 (paper square = 1 cm). This specimen shares some traits with stage 3 lambs (e.g. very marked face/snout truncation, left and right muscle asymmetries, ‘nasal proboscis’ mainly situated on right side of head) but is more defective than stage 3 lambs because only one of the three small anterior facial snout muscles in present in both sides of the head - the levator labii superioris. For more details see text and SI1 Table 1.

Individuals such as specimens 9 and 4 display what we consider to be myological defective stage 3 (SI1 Table 1). This is because the marked truncation of the face/snout is associated with a peculiar, asymmetrical configuration of the anterior facial muscles: on the left side of each of these specimens the levator anguli oris facialis, levator labii superioris and dilatator naris lateralis were more separated than on the right side, where only two main structures can be seen, one going markedly anterodorsally to the dorsal part of the ‘nasal proboscis’, and the other one going to the main body of this proboscis. That is, apparently there was no distinct, separate levator anguli oris facialis going mainly to the upper lip on the right side of these specimens (SI1 Table 1). Specimen 122B shares some traits with these stage 3 lambs, including its very marked face/snout truncation, the presence of some left-right muscle asymmetries, and the fact that it displays a ‘nasal proboscis’ that is mainly situated on the right side of the head (Fig. 4). However, this specimen 122B is more defective than stage lambs displaying a myological stage 3 because more muscles are missing: both the right and left sides of its head have only one of the three small anterior facial snout muscles - the levator labii superioris (i.e. the levator anguli oris facialis and dilatator naris lateralis are seemingly missing) (Fig. 4). Also, the extraocular muscle inferioris rectus was abnormal in this specimen 122B because it was fused with its counterpart in the midline (see SI1 Table 1). However, contrary to specimens of myological stage 4 (see below), in specimen 122B the levator labii superioris muscles of the two sides of the head do not form a circular, constrictor-like complex (Fig. 4). That is why we consider that this specimen 122B displays a stage 3.5 of myological defects (Fig. 4).

**Figure 5:**
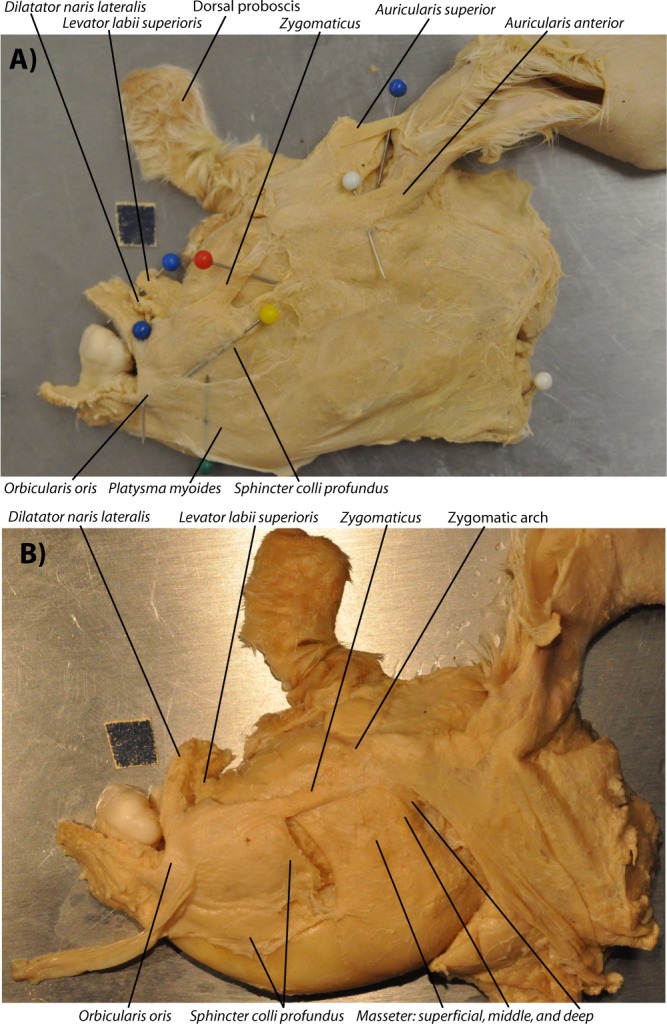
Lateral view of head muscles of ‘myological stage 4’ newborn lambs. Typical configuration of more superficial (**A**) and deep (**B**) facial and masticatory muscles of myological stage 4 newborn lambs dissected by us (paper square = 1 cm), as seen here on the left side of specimen 199. Basically, at this stage the truncation of the head is very extreme, there is almost always a well-developed dorsal (above the region of the eye) proboscis, and usually the levator anguli oris facialis and/or the dilatator naris lateralis are fused or missing, and the levator labii superioris is continuous in the midline with that of the other side of the head, the two muscles forming a circular, constrictor-like muscle complex. For more details see text and SI1 Table 1.

In individuals such as specimen 199 (Fig. 5), which osteologically correspond to or are at least very similar to the osteological cyclopic stage sensu Evans *et al*.^5^ (osteological stage 4 sensu the present work: Fig. 1), the face/snout is even more truncated, the two joined eyes share a single orbital cavity. In this specimen 199 apart from the ‘nasal proboscis’ often found in myological stage 3 ad 3.5 newborn lambs (see Fig. 4) there is also a proboscis above the eye region - i.e. a ‘dorsal proboscis’ sensu the present work, which is similar to that often present in humans with cyclopia. In this specimen the levator anguli oris facialis is either not present or is fused with the dilatator naris lateralis, and the levator labii superioris is continuous in the midline with that of the other side of the head, the two muscles forming a circular, constrictor-like muscle complex where the upper region of the snout should normally be present (Fig. 5; for more details see SI1 Table 1). A similar example was seen in individual 3, which is also a myological stage 4 specimen (SI1 Table 1). The face/snout are markedly truncated and the skull is cyclopic, there is both a ‘dorsal proboscis’ and a ‘nasal proboscis’, and the levator labii superioris was similar to that described for specimen 199. The main difference is that on the right side, just below this muscle, originated (also from the facial tuber) a single muscle that went exclusively to the ‘nasal proboscis’: this muscle thus mainly corresponds to the dilatator naris lateralis, the apparent absence of the levator anguli oris facialis being likely related to the fact that there was no upper lip at all. The eyes had all the normal muscles except the medial rectus that seemed to be absent in both sides, as there was in fact no space for these muscles between the joint eyes. In addition, the inferior rectus seemed to be a wide muscle extending all the way to the midline and thus probably meeting with the counterpart at the midline.

An even more extreme configuration is seen in specimen 993 (which somewhat resembles a case of human ethmocephaly), in which there is no nose/upper jaw/nasal proboscis at all (SI1 Table 1). That is, above the region of the lower jaw/lips lies only fatty tissue, and the muscles that should lie in that region (sphincter colli profundus pars palpebralis, nasalis, levator labii superioris alaeque nasi, levator anguli oris facialis and dilatator naris lateralis) are all missing, with exception to the levator labii superioris. Interestingly, all the other head muscles were present and basically had their normal attachments, except the fact that a) the masseter was mainly constituted by a single bundle, i.e. its 3 normal heads were deeply fused to each other; b) the medial rectus was missing; and c) as in specimen 199 the levator labii superioris is continuous in the midline with that of the other side of the head, forming a circular, constrictor-like muscle complex. In fact, a distinctive feature seen in all stage 4 newborn lambs is that the extraocular muscle medial rectus was missing (SI1 Table 1). In addition, in some (e.g. specimen 3) but not all stage 4 lambs the inferior rectus was also fused with its counterpart in the midline, as was also the case in the stage 3.5 specimen 122B described above.

**Figure 6:**
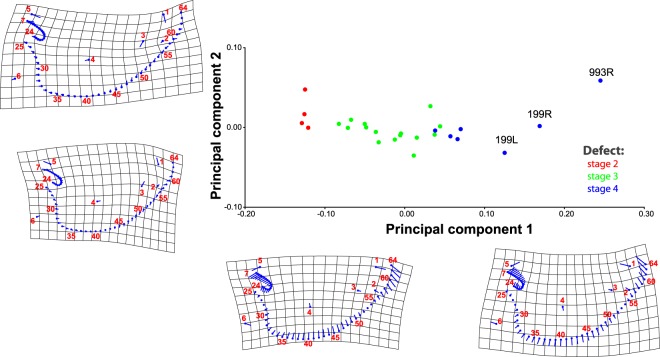
Principal component analysis results from our morphometric analysis, for lower jaw. For the lower jaw, it is possible to discern more or less clear-cut morphometric differences that match with our myological classification of myological stages 2, 3 and 4. For more details, see SI2.

Regarding our morphometric analyses, from the detailed results that we provided in SI2 (see also Fig. 6), two are particularly interesting. Firstly, they quantitatively confirm that there is some, but importantly not a one-to-one, match between the level of osteological defects sensu Evans *et al*. (Fig. 1) and the level of myological defects seen in our dissections. For instance, some newborn lambs that are osteologically stage 3 (*cebocephalic* sensu Evans *et al*.^5^) do have all the head muscles (stage 2 myologically), while others are missing one muscle, which is usually a small anterior snout muscle that is missing on the right side of the head (myological stage 3 described above). The specimens 6 (Fig. 3), 2 and 5 are examples of specimens that would be coded as being close to or in osteological stage 3 but that are advanced myological stage 2 because they have all the muscles that are normally present in newborn lambs. This means, of course, that there is in fact a continuum between each ‘defective stage’, both osteologically and myologically, as our own terminology recognizes when we refer to a ‘mild myologically defective’ stage 2 (Fig. 2) vs more a ‘more marked myologically defective’ stage 2 (Fig. 3). A second interesting result of the morphometric analyses is that they also provide quantitative support for this slight osteological-myological mismatch because they show that, contrarily to the soft tissue left-right asymmetries that are often present in myological stages 3 to 4, there is no marked directional asymmetry concerning osteological structures (for more details, see SI2).

## Discussion

Based on our results, we can define four major stages between the normal (stage 1) and cyclopic condition (stage 4) in terms of the configuration of the head muscles (Figs 2–5) that have some - but not a one-to-one - correspondence to the four stages of defects based exclusively on osteological analyses (Fig. 1). For instance, the level of defects seen in the head muscular system as a whole is more mild that that seen in the head skeletal system. An example is that in the typical muscle configuration of stage 2 newborn lambs (between *normal* and *cebocephalic* sensu Evans *et al*.^5^) all head muscles are usually present and have a mainly normal configuration (Fig. 2). Such a pattern was, interestingly, also seen in the very few muscular descriptions of humans with cebocephalic skulls: despite the severe osteological defects, in these human individuals all the normal extraocular and facial muscles were present and seemingly had a normal configuration (reviewed in^6^).

In more extreme configurations within myological stage 2, i.e. more similar to myological stage 3 than to stage 1, the head is more highly truncated and the facial tuber lies very posteriorly at the level below the region of the eyes. Therefore, the direction of the fibers of muscles attached to this tuber, such as the sphincter colli pars palpebralis, are different from normal but all muscles are still usually present despite the remarkable truncation of the skull (see Fig. 3).

In myological stage 3 newborn lambs the truncation of the head is even more marked than in stage 2, and there is often a well-developed ‘nasal proboscis’ that lies mainly on the right side of the head, and usually there are 3 small muscles on the snout region (dilatator naris lateralis, levator labii superioris, and levator anguli oris facialis) on the left side but only two on the right side. As noted above, another interesting result of our work is that, contrarily to such left-right myological asymmetries, there is no marked directional asymmetry in the skull of specimens belonging to this or any other defective stages, as revealed by our gross anatomical and morphometric analyses (see SI2). At least some eye muscles might be affected at this myological stage 3 (SI1 Table 1).

The muscle configuration seen in myological stage 4 (Fig. 5) cyclopic newborn lambs is even more extreme than in myological stages 3 and 3.5 (Fig. 4). As noted above, myological stage 4 specimens often have, apart from the ‘nasal proboscis’ often seen in myological stages 3 and 3.5, a well-developed ‘dorsal proboscis’ similar to that usually found in cyclopic humans (Fig. 5). In this myological stage 4 various anterior facial muscles are usually missing and the levator labii superioris is continuous in the midline with that of the other side of the head, the two muscles forming a circular, constrictor-like muscle complex as noted above (Fig. 5). As also noted above, another distinctive feature displayed by all myological stage 4 lambs is the absence of the extraocular muscle medial rectus; also in some but not all these lambs the inferior rectus was fused with its counterpart in the midline, a condition also seen in the stage 3.5 specimen 122B (SI1 Table 1).

These observations, and the comparisons with humans, strongly support Alberch’s ill-named “logic of monsters” because, as predicted by Alberch, the muscle defects seen in distantly related taxa tend to be very similar as a byproduct of strong developmental and/or topological constraints^13^. Alberch’s theory states that the variation/defects in normal/abnormal individuals of a certain taxon (e.g. modern humans) mirror the fixed normal phenotype observed in members of other taxa^13^. This predictable - ‘logic’ - parallel is due to regulation of a constrained ontogenetic program (e.g. a set of epigenetic and/or genetic relations), in the sense that these internal relations constrain the possible variations upon which selection can function, and thus the possible anatomical outcomes^13^. For recent, detailed discussion on the links between Alberch’s “logic of monsters” and Evo-Devo-Path, see Diogo *et al*.^3,14^.

**Figure 7:**
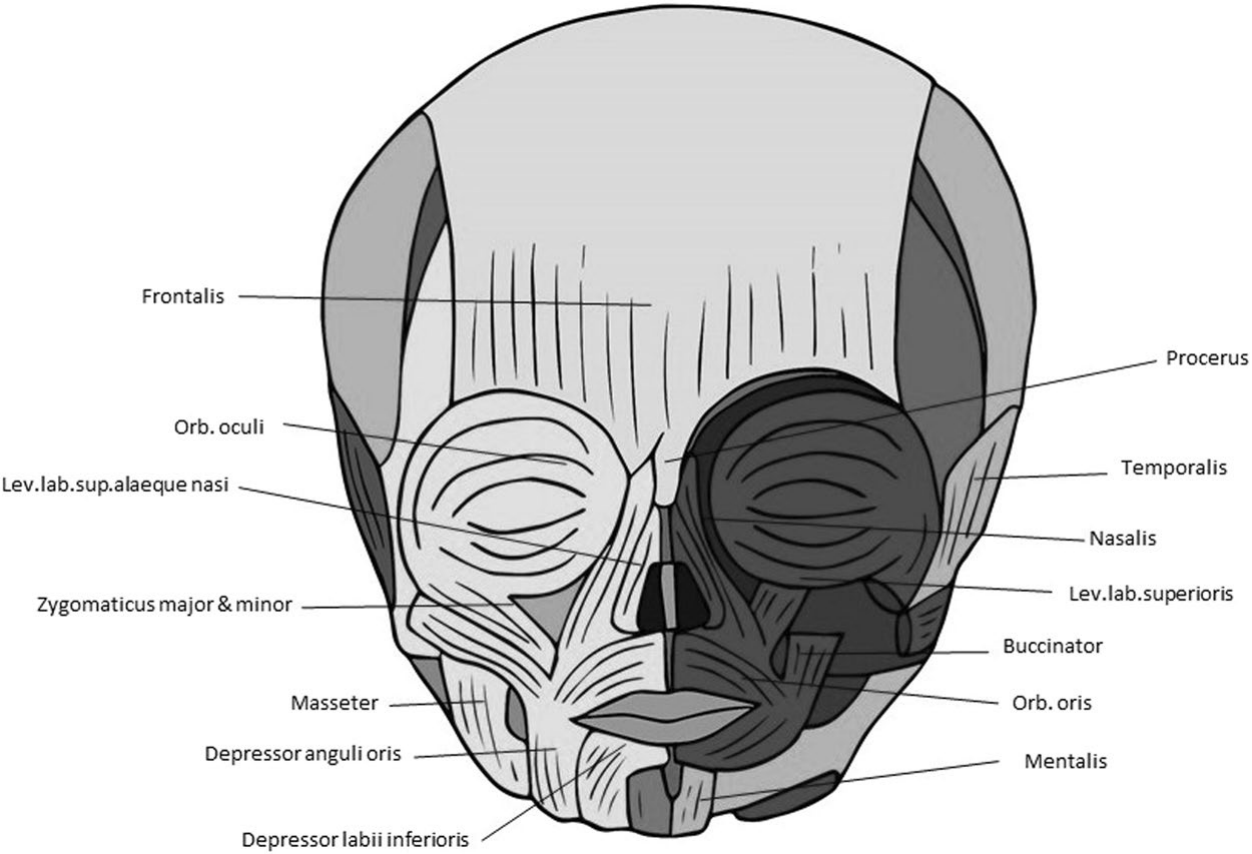
Head muscles in normal human newborn. Anterior view showing head musculature in a normal human newborn (above) (modified from Smith *et al*.^5^). For more details, see text and Smith *et al*.^5^.

**Figure 8:**
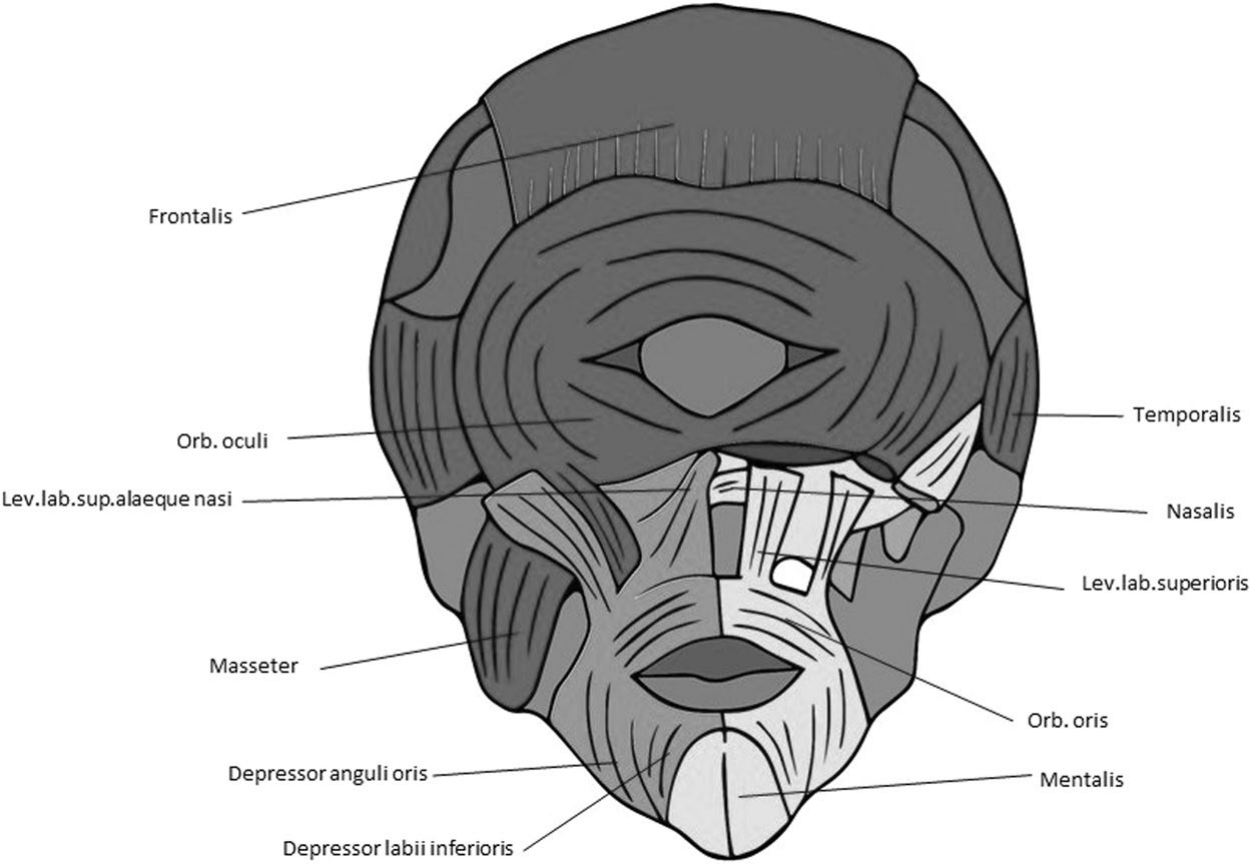
Head muscles in human cyclopia. Anterior view showing head musculature in a cyclopic human fetus with trisomy-18 (modified from Smith *et al*.^5^). For more details, see text and Smith *et al*.^5^.

For instance, the muscle defects that we found in newborn lambs are very similar to those found in late fetal/newborn humans with cyclopia (see Figs 7, 8). That is, apart from the obvious fact that cyclopic lambs and humans with cyclopia share a single orbit, due to strong developmental constraints many other traits that seem to be mainly byproducts of the defects leading to cyclopia are shared by lambs and humans, as e.g. the presence of a ‘dorsal proboscis’ (because the nose cannot physically migrate ventrally: for a recent review on this subject, see^4^). The similarities between the muscles of the myological stage 4 lambs and those found in cyclopic humans are in fact striking. In both cases the more posterior head muscles are not affected or are just slightly affected, the more affected ones being those of the eye and those lying in the anterior region between the orbit and the mouth (compare Figs 5 and 7, 8). Moreover, muscles that are normally separated by the nose (in humans)/snout (in lambs), or that normally just contact their counterparts via a few fibers in the midline, invade the region of the missing nose/snout in cyclops and form together with their counterparts a constrictor-like, circular complex muscle structure (compare Figs 5, 7 and 8). In fact, these comparisons support the idea that these are homoplasic (i.e. non-homologous) similarities that are mainly byproducts of strong developmental/topological constraints as predicted by Alberch. This is because the facial muscles sharing such similar configurations in humans and lambs are not the same. In lambs this happens usually to the levator labii superioris (Fig. 5) while in humans it happens to the nasalis and/or levator labii superioris alaeque nasi (Fig. 8). That is, these similar configurations seem to be related to the fact that, as these facial muscles cannot go to the osteological structures to which they normally attach, they go to the place that lies topologically more near to the missing structures.

In this sense, our observations and comparisons also support the idea that the facial muscles of mammals reverted to a plesiomorphic ‘nearest neighbor’ type of muscle-bone attachment typically seen in non-vertebrate animals and still found in vertebrate limbs - not in vertebrate heads. This idea was originally proposed from the observation that non-pentadactyl limbs of wildtype taxa such as salamanders, frogs, crocodilians and chickens, and humans with congenital malformations, exhibit a surprisingly consistent “*nearest neighbor*” pattern^2–4^. That is, the identity and attachments the muscles of these non-pentadactyl limbs is seemingly mainly linked to the topological (physical) position of the digits to which the muscles are inserted, and not to the primordium, number, or homeotic identity of the digits. Such a “nearest neighbor” model of muscle-tendon-skeleton links within the limbs is unlike models proposed for the neck and head muscles/tendons/skeleton derived from the branchial arches (i.e., 1st or mandibular arch, 2nd or hyoid arch, and more posterior arches). In fact, Köntges & Lumsden’s^15^ works with rhombomeric quail-to-chick grafts revealed that each rhombomeric neural crest cell population remains consistent during development: there is a rhombomere-specific matching of tendons and their attachments for most head and neck muscles. Köntges & Lumsden model is, therefore, more of a “*find and seek*” type, because the bone/tendon/muscle associations are mainly linked to the arch (homeotic) identity, and not to topological position: tendons of muscles of a certain arch in general insert to skeletal structures derived from that same arch. Köntges & Lumsden model can thus be also very useful in the sense that it provides a possible mechanistic explanation of why the level of defects seen in the head muscular system as a whole is more mild that that seen in the head skeletal system, within the holoprosencephalic lambs analyzed by us. This is because according to that model it is predicted that even if skeletal structures are highly deformed, the presence and attachments of muscles are not so severely affected; this seems also to be the reason why the muscular system is in general more conservative in evolution and so appropriate to study the phylogenetic relationships among higher taxa (reviewed in^16–22^).

However, most facial expression muscles of mammals, which are second (hyoid) arch muscles, insert onto structures *not* derived from the second arch, such as the skin, and even postcranial skeletal structures^3^. In fact, Prunotto *et al*.^23^ have shown that with regard to C-met mutations the muscles of facial expression share more similarities with the hypobranchial (tongue and infrahyoid) and limb migratory muscles, which are derived from the somites and that usually migrate far away from their origin, being missing in animals with C-met mutations, contrary to the other true (branchiomeric) muscles of the head and neck. Moreover, investigations of humans with congenital malformations also sustain the hypothesis that facial expression muscles act more like the muscles of the limbs than like other branchiomeric muscles. This is because they seem to display a “*nearest neighbor*” - rather than a “*find and seek*” - model of muscle/tendon/bone association. One of the illustrative examples that has been used to support this idea is precisely the trisomy 18 cylopic fetus shown in Fig. 8^3,4^. This is because this fetus lacks a proboscis, and any other structure that would correspond to an external nose: yet, the facial expression muscle nasalis that normally attaches onto the nose is still present, attaching to a prominence of the maxilla lying in the region where the nose is usually situated^3,4^.

Coming back to the similarities between cyclopic lambs and cyclopic humans, a further example of such resemblances is the usual absence of the rectus medialis (humans condition reviewed in e.g.^4,6,24^). This phenotype shared by cyclopic lambs and humans seems again to be simply a byproduct of the fact that there is no space between the eyes to be occupied by the left and right rectus medialis muscles that are present in the normal phenotype. Similarly, in both humans and lambs with cyclopia the rectus inferioris is sometimes fused with its counterpart at the midline (humans condition described in e.g.^4,6,24,25^). Within the relatively scarce literature on soft-tissue anomalies found in other holoprosencenphalic non-human mammals another illustrative example of a similarity shared with the abnormal lambs dissected by us was the presence of a ‘nasal proboscis’ (see, e.g., Fig. 4). This ‘nasal proboscis’ - which is often present in myological stages 3, 3.5 and 4 newborn lambs but not present in holoprosencenphalic humans (and which should thus not be confused with the ‘dorsal proboscis’ that is often present in cyclopic animals, including humans) - is in fact strikingly similar to that often seen in holoprosencenphalic piglets (‘snout-wedge on upper limb’ sensu Fisher *et al*.^26^). Moreover, as observed in some of the holoprosencenphalic newborn lambs dissected by us, holoprosencenphalic piglets often have both a ‘dorsal proboscis’ and a ‘nasal proboscis’.

According to Fisher *et al*.’s descriptions, in the holoprosencenphalic piglets analysed by them this ‘nasal proboscis’ was covered with the “typical glabrous epithelium” that was also found in the ‘dorsal proboscis’, and that “had vibrissae, connective tissue, and hypodermis resembling that of a normal snout; relatively normal-appearing facial muscles were attached to (it) however no external nares and no cavities were present”^16:139–140^. These authors suggested two possible origins for these ‘nasal proboscis’ of the holoprosencephalic piglets: “in cases lacking any nasal structures, it may have developed from the region of fusion of the maxillary prominences; this implies that the nasomedial and nasolateral prominences failed to develop and that the development of the wedge would be determined by its surrounding tissue since the maxillary prominences do not normally form glabrous epithelium”^16:144^. Alternatively, “in cases with a (dorsal) proboscis, the medial movement of the eyes may have ‘pinched’ the nasal prominences such that some tissue was caught both ventral and dorsal to the fusing eyes thus represents an embryological remnant of either the nasomedial or nasolateral prominences”^16:144^. They argued that the presence of a large protruding snout in the normal swine phenotype might explain why holoprosencephalic pigs often have such a ‘nasal proboscis’, contrarily to holoprosencephaly humans. This idea seems to be supported by the fact that in lambs, which have normally large protruding snouts like piglets do, holoprosencephaly is often also associated with the presence of such a ‘nasal proboscis’. That is, once again, as predicted in Alberch’s “logic of monsters”, this shared similarity in holoprosencephalic lambs and piglets does not seem to be related to the occurrence of the same genetic condition/syndrome (as the same phenotype is not seen in holoprosencephalic humans), but seems instead simply to be the byproduct of the defects leading to this condition, occurring in a topologically similar type of face (i.e., a large protruding snout). That is, as predicted by Alberch, these similarities seem to be byproducts of developmental/topological constraints.

Further comparative studies, including developmental and molecular works, are needed to further test these ideas, including Fisher *et al*.’s ideas about the formation of a ‘nasal’ proboscis in taxa that normally have large protruding snouts such as piglets and lambs. In fact, a major goal of the present work is to pave the way for, and stimulate researchers to undertake, such evo-devo-path studies. In particular, the outcomes of the present work and particularly of the comparisons between lambs, humans, and other taxa stress the point that the studies of skeletal and particularly of soft-tissue defects in these taxa are not merely a curiosity, a relict of museums of anatomy or of ‘rarities’. Instead, the detailed study of such defects is particularly important to link the study of normal and abnormal development, evolution, and pathologies, as is being stressed by the increasing number of authors currently publishing work that can be considered to be part of this evo-devo-path field^1–4,14^. It is precisely hoped that this paper will further stimulate the development of this field, and in particular to link fields such as comparative anatomy, zoology, evolutionary developmental biology, developmental biology, veterinary, pathology, and medicine in general.

## Materials and Methods

We dissected six sides of heads of normal newborn lambs - i.e. sheep less than 1 year old - fixed in ethanol (provided by Howard Evans, from his private collection at Cornell Univ.) and normal adult sheep (fresh, provided by a local DC slaughterhouse), as well as 27 sides of abnormal newborn lamb fixed in ethanol (also provided by Howard Evans) (for more details, see SI1 Table 1). The abnormal newborn lambs were collected during various years by Howard Evans and colleagues to illustrate various degrees of skull osteological abnormalities, from mild defects (stage 2 sensu the present work, stage 1 being a normal phenotype) to a *cebocephalic* configuration (stage 3 sensu the present work), and then from a configuration between *cebocephalic* and *cyclopic* (stage 3.5 sensu the present work) to cyclopic (stage 4 sensu the present work): see Evans *et al*.^6^ and Fig. 1. By using Evans *et al*.^5^ classification, and adapting it to the osteological stages 1, 2, 3, and 4 sensu the present work, we were able to infer the osteological defective stage to each specimen dissected by us belonged to, particularly with the help of CT scans (not shown) done at the radiology department of Howard University *before* the dissections were done. The osteological information obtained in these scans and the different stages assigned to them confirmed *a posteriori* by removal of all the soft tissues of each side of the head of the specimens dissected by us after the dissections of the muscles were done. It should be noted that no autopsies of the brains were carried out.

The data obtained from the CT scans, as well as the detailed osteological information previous compiled by Evans *et al*.^5^ for each osteological stage of defect of the newborn lambs analyzed by us were crucial to help discern the osteological structures to which each muscle dissected by us attached to, in order to discern if the configuration of each muscle was normal or not (see SI1 Table 1). All the muscle dissections were done under magnification using a Nikon SMZ1500 stereomicroscope and photographed with a Nikon DS-Fi1 digital microscope camera or with a Nikon D90 camera with an AF-S Micro Nikon 60-mm lens. The myological nomenclature used in this study follows those of our previous works^27–29^ (see SI1 Table 1). In order to facilitate comparisons with terminologies used by other authors working with sheep/lambs or other domestic animals, synonymies between the names used by us and those used by other authors (e.g.^28,29^) are also given in SI1 Table 1. For the morphometric analysis of all the left and right sides of the skull of each abnormal specimen dissected by us, we first removed all the soft tissues of those specimens as noted above, and then used landmarks that are consistently used for morphometric analysis of mammalian skulls, in order to run our analyses, which included Proctustes ANOVA; more details on the morphometric methods used by us, as well details concerning the results obtained from them, are summarized in SI2.

## Supplementary information

Supplementary info
