# Supplementary material for: Musculoskeletal study of cebocephalic and cyclopic lamb heads illuminates links between normal and abnormal development, evolution and human pathologies
Source: Sci Rep. 2019 Jan 30;9:991. doi: 10.1038/s41598-018-37735-9 (PMC6353885; doi:10.1038/s41598-018-37735-9)
Supplement: Supplementary file 1 — Supplementary info [file 41598_2018_37735_MOESM1_ESM.pdf]

Musculoskeletal study of cebocephalic and cyclopic lamb heads illuminates links between normal and abnormal development, evolution and human pathologies

Authors: Rui Diogo, Daria Razmadze, Natalia Siomava, Nora Douglas, Jose S. M. Fuentes, Andre Duerinckx

Supplementary Information 1, incluinding SI1Tab.1

**SI1Tab1: results obtained from specimens dissected by us (R=right; L=left; anomalies in red)**	**Normal (stage 1) configuration seen in 6 sides of normal newborn lamb and adult sheep specimens**	**Abnormal specimen 6L (dissected by DR) and 6R (dissected by RD; stage 2)**	**Abnormal specimens 12L (dissected by DR) and 16R (dissected by ND; stage 2)**	**Abnormal specimens 2R, 2L, 5R, 5L (dissected by RD; stage 2)**	**Abnormal specimens 10L and 10R and 16L (dissected by RD; stage 2)**	**Abnormal specimen 65L and 65R (dissected by DR; stage 3)**	**Abnormal specimen 122AL and 122AR (dissected by DR; stage 3)**	**Abnormal specimen 4L and 4R (dissected by RD; stage 3)**	**Abnormal specimen 9L and 9R (dissected by RD; stage 3)**	**Abnormal specimen 122BR, 122BL (dissected by NS; stage 3.5)**	**Abnormal specimen 3L (dissected by ND) and 3R (dissected by RD; stage 4)**	**Abnormal specimen 11L (dissected by ND; stage 4)**	**Abnormal specimen 199R and 199L (dissected by DR; stage 4)**	**Abnormal specimen 993R (dissected by RD; stage 4)**
Platysma myoides	Mainly from skin of the neck/pectoral region to skin and muscles of mouth region	Mainly normal	Mainly normal	Mainly normal	Mainly normal	Mainly normal	Mainly normal	Mainly normal	Mainly normal	Mainly normal	Mainly normal	Mainly normal	Mainly normal	Mainly normal
Platysma cervicale	Mainly from skin of temporal/nuchal region to skin and muscles of mouth region	Mainly normal	Mainly normal	Mainly normal	Mainly normal	Mainly normal	Mainly normal	Mainly normal	Mainly normal	Mainly normal	Mainly normal	Mainly normal	Mainly normal	Mainly normal
Retractor anguli oculi medius profundus (= 'Posterior portion of malaris' or 'depressor palpebrae inferioris' sensu May 1964)	Mainly from deep fascia covering anterior portion of masseter, plus orbicularis oculi, to fascia of lower eyelid	Mainly normal	Mainly normal	Mainly normal	Mainly normal	Mainly normal	Mainly normal	Abnormal: seems to be not present as a separated muscle	Mainly normal	Mainly normal	Mainly normal	Mainly normal	Mainly normal	Mainly normal
Sphincter colli profundus proprius	Mainly deep to platysma cervicale and platysma myoides, gives rise to sphincter colli profundus pars palpebralis (note: it is not continuous with sphincter colli profundus pars palpebralis)	Mainly normal	Mainly normal	Mainly normal	Mainly normal	Mainly normal	Mainly normal	Mainly normal	Mainly normal	Mainly normal	Mainly normal	Mainly normal	Mainly normal	Mainly normal
Sphincter colli profundus pars palpebralis (= 'Anterior portion f malaris' or 'levator buccalis' sensu May 1964)	Mainly from buccinatorius to lacrimal bone	Mainly normal	Mainly normal	Abnormal, because due to the truncation of the snout, it runs antero-dorsally (as it originates mainly from the	Abnormal, because due to the truncation of the snout, it runs antero-dorsally (as it originates mainly from the	Abnormal, because due to the truncation of the snout, it runs antero-dorsally (as it originates mainly from the	On the right side, there are two separate parts; one of them more rostral: it originates from	Abnormal, because due to the truncation of the snout, it runs antero-dorsally (as it originates mainly from the	Abnormal, because due to the truncation of the snout, it runs antero-dorsally (as it originates mainly from the region of	Abnormal, because due to the truncation of the snout, it runs antero-dorsally (as it originates mainly from the	Abnormal, because due to the truncation of the snout, it runs antero-dorsally (as it originates mainly from the	Abnormal, because due to the truncation of the snout, it runs antero-dorsally (as it originates mainly from the	Seemingly missing	Seemingly missing

				region of the facial tuber, which is markedly more posterior than normally) to reach the eye region, and not posterodorsally as normally; that is, basically the size and direction of the fibers changes, but the attachments are mainly normal	region of the facial tuber, which is markedly more posterior than normally) to reach the eye region, and not posterodorsally as normally; that is, basically the size and direction of the fibers changes, but the attachments are mainly normal	region of the facial tuber, which is markedly more posterior than normally) to reach the eye region, and not posterodorsally as normally; that is, basically the size and direction of the fibers changes, but the attachments are mainly normal	the superior part of buccinator area, goes diagonally forward and inserts directly in front of the orbita. The other part lies more superficial, originate from the posterior area of the cheek from the sphincter colli profundus, and inserts onto the orbicularis oculi in the area directly under the orbita	region of the facial tuber, which is markedly more posterior than normally) to reach the eye region, and not posterodorsally as normally; that is, basically the size and direction of the fibers changes, but the attachments are mainly normal	the facial tuber, which is markedly more posterior than normally) to reach the eye region, and not posterodorsally as normally; that is, basically the size and direction of the fibers changes, but the attachments are mainly normal	region of the facial tuber, which is markedly more posterior than normally) to reach the eye region, and not posterodorsally as normally; that is, basically the size and direction of the fibers changes, but the attachments are mainly normal, although on the left side of the head the dorsal attachment was more anterior to the eye region (where it normally attaches in normal newborn lambs)	region of the facial tuber, which is markedly more posterior than normally) to reach the eye region, and not posterodorsally as normally; that is, basically the size and direction of the fibers changes, but the attachments are mainly normal	region of the facial tuber, which is markedly more posterior than normally) to reach the eye region, and not posterodorsally as normally; that is, basically the size and direction of the fibers changes, but the attachments are mainly normal		
Sphincter colli profundus pars auricularis (= 'Parotido-auricularis' or 'Auricularis inferior et depressor auriculae' sensu May 1964) (Note: the mandibuloauricular is, often named 'styloauricularis' in ruminants, is absent in goats/sheep)	Deep to platysma cervicale, inserts dorsally to region of the ear	Mainly normal	Mainly normal	Mainly normal	Mainly normal	Mainly normal	Mainly normal	Mainly normal	Mainly normal	Mainly normal	Mainly normal	Mainly normal	Mainly normal	Mainly normal
Zygomaticus	From zygomatic bone to angle of mouth/upper lip	Mainly normal	Mainly normal	Mainly normal	Mainly normal	Mainly normal	Mainly normal	Mainly normal	Mainly normal	Mainly normal	Mainly normal	Mainly normal	Mainly normal	Mainly normal
Levator labii superioris alaeque nasi (= 'Levator nasolabialis' sensu	From skin if frontal and nasal regions, and from orbicularis oculi,	Mainly normal	Mainly normal	Mainly normal	Mainly normal	Mainly normal	Mainly normal	Mainly normal	Mainly normal	Mainly normal	Mainly normal	Mainly normal	Mainly normal	Seemingly missing

May 1964)	meeting its counterpart at the midline, to nostril and upper lip													
Nasalis (= 'Dilatator naris apicalis' or 'Transversus nasi' sensu May 1964)	From premaxilla ('incisive bone') tonostril, meeting its counterpart at a median raphe	Not analyzed	Not analyzed	Not analyzed	Not analyzed	Not analyzed	Not analyzed	Not analyzed	Not analyzed	Not analyzed	Not analyzed	Not analyzed	Not analyzed	Seemingly missing
Dilatator naris lateralis (= 'Caninus' sensu Getty 1975, who confused the identity of the true caninus, which he named 'depressor labii maxillaris', see below) (Note: the dilatator naris alaris is seemingly poorly developed in the ox (Getty 1975) and very reduced/absent in sheep, as it was not described as a separate muscle by May 1964, nor found by us; according to Getty 1975 in ruminants it is usually fused with the levator anguli oris facialis, levator labii superioris alaeque nasi, levator labii superioris and dilatator naris lateralis, originating from the premaxilla and lateral nasal cartilages, and inserting onto the nostril)	From facial tuber of maxilla to nostril	Wide but short because of truncation of snout	Mainly normal	Mainly normal	Mainly normal	Not analyzed	Not analyzed	On the left side it originated as usual from facial tuber, but on the right side it originated anteriorly to the facial tuber; on both sides it attached mainly to the 'proboscis'; moreover, it was deeply fused with the levator anguli oris facialis and with the levator labii superioris on the right side, only two of these three muscles being really seen on that right side	On the right side only a muscle was present where the dilatator naris lateralis and levator anguli oris facialis are usually present, while on the left side both muscles were present as distinct structures	Seemingly missing	On the right side only a muscle was present where the dilatator naris lateralis and levator anguli oris facialis are usually present; it was difficult to analyze these muscles on the left side	Seemingly missing, or at least not clearly present as separate muscle/highly modified	On both the left and right there was seemingly only a muscle where normally lie the levator anguli oris facialis and the dilatator naris lateralis	Seemingly missing
Levator labii superioris (= 'Levator labii	From facial tuber of maxilla toupper lip and	Wide but short because of truncation of	Mainly normal	Wide but short because of truncation of	Mainly normal	Wide but short because of truncation of	Wide but short because of truncation of	On the left side it originated as usual from facial	Wide but short because of truncation of snout	Wide but short because of truncation of	Very peculiar, very well developed,	Seemingly missing, or at least not clearly	Very peculiar, very well developed,	Very peculiar, running from the facial tuber

maxillaris' or 'Levator labii superioris proprius' sensu May 1964)	skin between nostrils, meeting its counterpart at the midline	snout		snout		snout	snout	tuber, but on the right side it originated anteriorly to the facial tuber; on both sides it attached mainly to the 'proboscis'; moreover, it was deeply fused with the levator anguli oris facialis and with the dilatator naris lateralis on the right side, only two of these three muscles being really seen on that right side		snout	running from the facial tuber almost completely dorsomedially to form a 'constrictor' that met its counterpart at the midline just above the nose, i.e. it did not go at all to the region of the nasal proboscis	present as separate muscle/highly modified	running from the facial tuber almost completely dorsomedially to form a 'constrictor' that met its counterpart at the midline just above the nose, i.e. it did not go at all to the region of the nasal proboscis	almost completely dorsomedially to form a 'constrictor' that met its counterpart at the midline just above the nose, i.e. it did not go at all to the region of the nasal proboscis
Levator anguli oris facialis (= 'Depressor labii maxillaris' or 'Depressor labii inferioris' sensu May 1964) (Note: this is clearly the true levator anguli oris facialis because it is the only one of the small muscles in the region that goes to the upper lip, and moreover is the more inferior and deep one, fusing with the buccinatorius)	From maxilla to nostril and upper lib	Mainly normal	Mainly normal	Mainly normal	Mainly normal	Not analyzed	Not analyzed	On the left side it originated as usual from facial tuber, but on the right side it originated anteriorly to the facial tuber; on both sides it attached mainly to the 'proboscis'; moreover, it was deeply fused with the dilatator naris lateralis and with the levator labii superioris on the right side, only two of these three muscles being really seen on that right side	On the right side only a muscle was present where the dilatator naris lateralis and levator anguli oris facialis are usually present, while on the left side both muscles were present as distinct structures	Seemingly missing	On the right side only a muscle was present where the dilatator naris lateralis and levator anguli oris facialis are usually present; it was difficult to analyze these muscles on the left side	Seemingly missing, or at least not clearly present as separate muscle/highly modified	On both the left and right there was seemingly only a muscle where normally lie the levator anguli oris facialis and the dilatator naris lateralis	Seemingly missing
Depressor labii inferioris ('Depressor labii mandibularis' sensu May 1964; note that the depressor anguli oris is not present as a separate, well-	From mandible to inferior lip and adjacent skin	Mainly normal	Mainly normal	Mainly normal	Mainly normal	Mainly normal	Mainly normal	Mainly normal	Mainly normal	Mainly normal	Mainly normal	Mainly normal	Mainly normal	Mainly normal

defined muscle)														
Orbicularis oris (note: the 'incisivus mandibularis/superior' and 'incisivus maxillaris/superior' sensu May 1964 are part of the orbicularis oris sensu the present work)	From superior and inferior lips to mandible and skin around mouth	Mainly normal	Mainly normal	Mainly normal	Mainly normal	Mainly normal	Mainly normal	Mainly normal	Mainly normal	Mainly normal	Mainly normal	Mainly normal	Mainly normal	Mainly normal
Buccinatorius	From the alveolar border of maxilla and mandible to the entire length of the cheek	Mainly normal	Mainly normal	Mainly normal	Mainly normal	Mainly normal	Mainly normal	Mainly normal	Mainly normal	Mainly normal	Mainly normal	Mainly normal	Mainly normal	Mainly normal
Mentalis	From body of mandible near symphysis toskin of chin region	Mainly normal	Mainly normal	Mainly normal	Mainly normal	Mainly normal	Mainly normal	Mainly normal	Mainly normal	Mainly normal	Mainly normal	Mainly normal	Mainly normal	Mainly normal
Orbicularis oculi	From palpebral ligament and lacrimal tubercle toskin of eyelid region	Mainly normal	Mainly normal	Mainly normal	Mainly normal	Mainly normal	Mainly normal	Mainly normal	Mainly normal	Mainly normal	Mainly normal	Mainly normal	Mainly normal	Mainly normal
Frontalis (note that the levator anguli oculi medialis, or 'corrugator supercilii', is not present in ruminants according to Getty 1975, and not in sheep according to May 1964)	From skin of frontal region to eye region and skin of the dorsal surface of forehead	Mainly normal	Mainly normal	Mainly normal	Mainly normal	Mainly normal	Mainly normal	Mainly normal	Mainly normal	Mainly normal	Mainly normal	Mainly normal	Mainly normal	Mainly normal
Zygomaticoauricularis (= 'Zygomatico-auricularis' sensu May 1964) (Note: we are not including in this study the numerous small facial muscles related to the movements of the ear, but our	From zygomatic arch to auricular cartilage	Mainly normal	Mainly normal	Mainly normal	Mainly normal	Mainly normal	Mainly normal	Mainly normal	Mainly normal	Mainly normal	Mainly normal	Mainly normal	Mainly normal	Mainly normal

Zygomaticoauricularis (= 'Zygomatico-auricularis' sensu May 1964) (Note: we are not including in this study the numerous small facial muscles related to the movements of the ear, but our dissections indicate that their presence, and attachments, were constant and similar to the normal phenotype in all abnormal specimens we analyzed, both the more and the less defective ones)	From zygomatic arch to auricular cartilage	Mainly normal	Mainly normal	Mainly normal	Mainly normal	Mainly normal	Mainly normal	Mainly normal	Mainly normal	Mainly normal	Mainly normal	Mainly normal	Mainly normal	Mainly normal
Levator palpebrae superioris	From pterygoid crest to palpebral fascia of superior eyelid	Mainly normal	Mainly normal	Mainly normal	Mainly normal	Mainly normal	Mainly normal	Mainly normal	Mainly normal	Mainly normal	Mainly normal	Mainly normal	Not analyzed	Mainly normal
Rectus superioris	From sphenoid bone to eye	Mainly normal	Mainly normal	Mainly normal	Mainly normal	Mainly normal	Mainly normal	Mainly normal	Mainly normal	Mainly normal	Mainly normal	Mainly normal	Not analyzed	Mainly normal
Rectus inferioris	From sphenoid bone to eye	Abnormal, fused with rectus medialis, although its attachments are as normal	Mainly normal	Mainly normal	Mainly normal	Not analyzed	Mainly normal	Mainly normal	Mainly normal	Inferior rectus seemed to be a wide muscle extending all the way to the midline and thus probably meeting with the counterpart at the midline, being possibly a single muscle for the two eyes as a whole	Inferior rectus seemed to be a wide muscle extending all the way to the midline and thus probably meeting with the counterpart at the midline, being possibly a single muscle for the two eyes as a whole	Mainly normal	Not analyzed	Mainly normal
Rectus lateralis	From sphenoid bone to eye	Mainly normal	Mainly normal	Mainly normal	Mainly normal	Not analyzed	Mainly normal	Mainly normal	Mainly normal	Mainly normal	Mainly normal	Mainly normal	Not analyzed	Mainly normal
Rectus medialis	From region of optic foramen to eye	Abnormal, fused with rectus inferioris, although its attachments are	Mainly normal	Mainly normal	Mainly normal	Not analyzed	Mainly normal	Mainly normal	Mainly normal	Mainly normal	Seemingly missing	Seemingly missing	Not analyzed	Missing

**Muscles of the ear**														
Zygomaticoauricularis (= 'Zygomatico-auricularis' sensu May 1964) (Note: we are not including in this study the numerous small facial muscles related to the movements of the ear, but our dissections indicate that their presence, and attachments, were constant and similar to the normal phenotype in all abnormal specimens we analyzed, both the more and the less defective ones)	From zygomatic arch to auricular cartilage	Mainly normal	Mainly normal	Mainly normal	Mainly normal	Mainly normal	Mainly normal	Mainly normal	Mainly normal	Mainly normal	Mainly normal	Mainly normal	Mainly normal	Mainly normal
Masseter	Superficial layer from facial tuber, maxilla and zygomatic arch portion of zygomatic bone; middle and deep layers from zygomatic arch, malar bone, and maxilla; superficial layer to caudal and ventral parts and to angle of mandible; middle layer to caudal and ventral borders and angle of ramus of mandible; deep layer to neck of ramus and articular surface of mandible, coronoid process,	Mainly normal	Mainly normal	Mainly normal	Mainly normal	Mainly normal	Mainly normal	Mainly normal	Mainly normal	Mainly normal	Mainly normal	Mainly normal	Mainly normal	Mainly normal

	and rostral part of ramus of mandible													
Temporalis	From termporal bone, frontal and temporal crests and zygomatic arch to coronoid process of mandible	Mainly normal	Mainly normal	Mainly normal	Mainly normal	Mainly normal	Mainly normal	Mainly normal	Mainly normal	Mainly normal	Mainly normal	Mainly normal	Mainly normal	Mainly normal
Pterygoideus medialis	From palatine bone, pterygoid bone and pterygoid process of basisphenoid bone, to medial surface of mandible	Mainly normal	Mainly normal	Mainly normal	Mainly normal	Mainly normal	Mainly normal	Mainly normal	Mainly normal	Mainly normal	Mainly normal	Mainly normal	Mainly normal	Mainly normal
Pterygoideus lateralis	Only one head; from pterygpoalatine fossa and pterygoid process of basisphenoid to medial surface of mandible and base of coronoid process	Mainly normal	Mainly normal	Mainly normal	Mainly normal	Mainly normal	Mainly normal	Mainly normal	Mainly normal	Mainly normal	Mainly normal	Mainly normal	Mainly normal	Mainly normal
Digastricus	It is crossed by the stylohyoideus, and the anterior and posterior digastric bellies are connected by thin intermediate tendon, which does not perforate the stylohyoideus; the anterior head of digastricus is deeply blended with mylohyoideus; digastricus as a whole runs from jugular ('paramastoid' sensu May 1964) process of	Mainly normal	Mainly normal	Mainly normal	Mainly normal	Mainly normal	Mainly normal	Mainly normal	Mainly normal	Mainly normal	Mainly normal	Mainly normal	Mainly normal	Mainly normal

	occipital bone to mandible													
Mylohyoideus	Has superficial and deep bundles in sheep according to May 1964; both bundles run from mylohyoid line of mandible to hyoid bone and to midline raphe	Mainly normal	Mainly normal	Mainly normal	Mainly normal	Mainly normal	Mainly normal	Mainly normal	Mainly normal	Mainly normal	Mainly normal	Mainly normal	Mainly normal	Mainly normal
Stylohyoideus	Has a long thin tendon of origin; from muscular angle of stylohyoid bone ('great cornu of hyoid bone' sensu May 1964) tobasihyoid bone ('body of hyoid bone' sensu May 1964)	Much shorter than normal, but basically same attachments	Mainly normal	Much shorter than normal, but basically same attachments	Much shorter than normal, but basically same attachments	Much shorter than normal, but basically same attachments	Much shorter than normal, but basically same attachments	Much shorter than normal, but basically same attachments	Much shorter than normal, but basically same attachments	Much shorter than normal, but basically same attachments	Much shorter than normal, but basically same attachments, and deeply blended with the posterior belly of the digastricus	Much shorter than normal, but basically same attachments	Much shorter than normal, but basically same attachments	Much shorter than normal, but basically same attachments
Jugulohyoideus (= 'Occipitohyoideus' sensu May 1964*)*	From jugular process of occipital bone tostylohyoid bone	Mainly normal	Mainly normal	Mainly normal	Mainly normal	Mainly normal	Mainly normal	Mainly normal	Mainly normal	Mainly normal	Mainly normal	Mainly normal	Mainly normal	Mainly normal
Geniohyoideus	Contacts with counterpart at midline; from mandible just near to symphysis, to basihyoid	Mainly normal	Mainly normal	Mainly normal	Mainly normal	Mainly normal	Mainly normal	Mainly normal	Mainly normal	Mainly normal	Mainly normal	Mainly normal	Mainly normal	Mainly normal
Ceratohyoideus	From ceratohyoid (and also stylohyoid according to May 1964) tothyrohyoid	Mainly normal	Mainly normal	Mainly normal	Mainly normal	Mainly normal	Mainly normal	Mainly normal	Mainly normal	Mainly normal	Mainly normal	Mainly normal	Mainly normal	Mainly normal
Hyoideus transversus	Connects ceratohyoid cartilages	Mainly normal	Mainly normal	Mainly normal	Mainly normal	Mainly normal	Mainly normal	Mainly normal	Mainly normal	Mainly normal	Mainly normal	Mainly normal	Mainly normal	Mainly normal
Styloglossus	From stylohyoid to tongue	Mainly normal	Mainly normal	Mainly normal	Mainly normal	Mainly normal	Mainly normal	Mainly normal	Mainly normal	Mainly normal	Mainly normal	Mainly normal	Mainly normal	Mainly normal
Hyoglossus	From basihyoid and lingual process of	Mainly normal	Mainly normal	Mainly normal	Mainly normal	Mainly normal	Mainly normal	Mainly normal	Mainly normal	Mainly normal	Mainly normal	Mainly normal	Mainly normal	Mainly normal

Hyoglossus	From basihyoid and lingual process of thyrohyoid (but from stylohyoid according to May 1964, what seems to be a mistake) to tongue	Mainly normal	Mainly normal	Mainly normal	Mainly normal	Mainly normal	Mainly normal	Mainly normal	Mainly normal	Mainly normal	Mainly normal	Mainly normal	Mainly normal	Mainly normal
Genioglossus	From mandible (chin) just near to symphysis, to tongue	Mainly normal	Mainly normal	Mainly normal	Mainly normal	Mainly normal	Mainly normal	Mainly normal	Mainly normal	Mainly normal	Mainly normal	Mainly normal	Mainly normal	Mainly normal
Hyoepiglotticus	Contacts its counterpart at midline; innervated by hypoglossal nerve, so seems to be hypobrachial muscle, not true laryngeal or pharyngeal muscle; runs from basihyoid to epiglottic cartilage	Not analyzed	Not analyzed	Not analyzed	Not analyzed	Not analyzed	Not analyzed	Not analyzed	Not analyzed	Not analyzed	Not analyzed	Not analyzed	Not analyzed	Not analyzed
Cricothyroideus	From posterior border and lateral surface of arch of cricoid cartilage tolateral surface of lamina of, and lateral surface of, thyroid cartilage	Mainly normal	Mainly normal	Mainly normal	Mainly normal	Mainly normal	Mainly normal	Mainly normal	Mainly normal	Mainly normal	Mainly normal	Mainly normal	Mainly normal	Mainly normal
Cricoarytenoideus posterior (= 'Cricoarytenoideus dorsalis')	From dorsal surface and median ridge of lamina of cricoid cartilage toarytenoid cartilage	Mainly normal	Mainly normal	Mainly normal	Mainly normal	Mainly normal	Mainly normal	Mainly normal	Mainly normal	Mainly normal	Mainly normal	Mainly normal	Mainly normal	Mainly normal
Cricoarytenoideus lateralis	From rostral border and lateral surface of arch of cricoid cartilage to muscular	Mainly normal	Mainly normal	Mainly normal	Mainly normal	Mainly normal	Mainly normal	Mainly normal	Mainly normal	Mainly normal	Mainly normal	Mainly normal	Mainly normal	Mainly normal

Arytenoideus (= 'Arytenoideus transversus')	Unpaired, from arytenoid cartilages to arytenoid cartilages	Mainly normal	Mainly normal	Mainly normal	Mainly normal	Mainly normal	Mainly normal	Mainly normal	Mainly normal	Mainly normal	Mainly normal	Mainly normal	Mainly normal	Mainly normal
Thyroarytenoideus	From thyroid cartilage to arytenoid cartilage	Mainly normal	Mainly normal	Mainly normal	Mainly normal	Mainly normal	Mainly normal	Mainly normal	Mainly normal	Mainly normal	Mainly normal	Mainly normal	Mainly normal	Mainly normal

Musculoskeletal study of cebocephalic and cyclopic lamb heads illuminates links between normal and abnormal development, evolution and human pathologies

Authors: Rui Diogo, Daria Razmadze, Natalia Siomava, Nora Douglas, Jose S. M. Fuentes, Andre Duerinckx

Supplementary Information 1, incluinding SI1Tab.1

**SI1Tab1: results obtained from specimens dissected by us (R=right; L=left; anomalies in red)**	**Normal (stage 1) configuration seen in 6 sides of normal newborn lamb and adult sheep specimens**	**Abnormal specimen 6L (dissected by DR) and 6R (dissected by RD; stage 2)**	**Abnormal specimens 12L (dissected by DR) and 16R (dissected by ND; stage 2)**	**Abnormal specimens 2R, 2L, 5R, 5L (dissected by RD; stage 2)**	**Abnormal specimens 10L and 10R and 16L (dissected by RD; stage 2)**	**Abnormal specimen 65L and 65R (dissected by DR; stage 3)**	**Abnormal specimen 122AL and 122AR (dissected by DR; stage 3)**	**Abnormal specimen 4L and 4R (dissected by RD; stage 3)**	**Abnormal specimen 9L and 9R (dissected by RD; stage 3)**	**Abnormal specimen 122BR, 122BL (dissected by NS; stage 3.5)**	**Abnormal specimen 3L (dissected by ND) and 3R (dissected by RD; stage 4)**	**Abnormal specimen 11L (dissected by ND; stage 4)**	**Abnormal specimen 199R and 199L (dissected by DR; stage 4)**	**Abnormal specimen 993R (dissected by RD; stage 4)**
Platysma myoides	Mainly from skin of the neck/pectoral region to skin and muscles of mouth region	Mainly normal	Mainly normal	Mainly normal	Mainly normal	Mainly normal	Mainly normal	Mainly normal	Mainly normal	Mainly normal	Mainly normal	Mainly normal	Mainly normal	Mainly normal
Platysma cervicale	Mainly from skin of temporal/nuchal region to skin and muscles of mouth region	Mainly normal	Mainly normal	Mainly normal	Mainly normal	Mainly normal	Mainly normal	Mainly normal	Mainly normal	Mainly normal	Mainly normal	Mainly normal	Mainly normal	Mainly normal
Retractor anguli oculi medius profundus (= 'Posterior portion of malaris' or 'depressor palpebrae inferioris' sensu May 1964)	Mainly from deep fascia covering anterior portion of masseter, plus orbicularis oculi, to fascia of lower eyelid	Mainly normal	Mainly normal	Mainly normal	Mainly normal	Mainly normal	Mainly normal	Abnormal: seems to be not present as a separated muscle	Mainly normal	Mainly normal	Mainly normal	Mainly normal	Mainly normal	Mainly normal
Sphincter colli profundus proprius	Mainly deep to platysma cervicale and platysma myoides, gives rise to sphincter colli profundus pars palpebralis (note: it is not continuous with sphincter colli profundus pars palpebralis)	Mainly normal	Mainly normal	Mainly normal	Mainly normal	Mainly normal	Mainly normal	Mainly normal	Mainly normal	Mainly normal	Mainly normal	Mainly normal	Mainly normal	Mainly normal
Sphincter colli profundus pars palpebralis (= 'Anterior portion f malaris' or 'levator buccalis' sensu May 1964)	Mainly from buccinatorius to lacrimal bone	Mainly normal	Mainly normal	Abnormal, because due to the truncation of the snout, it runs antero-dorsally (as it originates mainly from the	Abnormal, because due to the truncation of the snout, it runs antero-dorsally (as it originates mainly from the	Abnormal, because due to the truncation of the snout, it runs antero-dorsally (as it originates mainly from the	On the right side, there are two separate parts; one of them more rostral: it originates from	Abnormal, because due to the truncation of the snout, it runs antero-dorsally (as it originates mainly from the	Abnormal, because due to the truncation of the snout, it runs antero-dorsally (as it originates mainly from the region of	Abnormal, because due to the truncation of the snout, it runs antero-dorsally (as it originates mainly from the	Abnormal, because due to the truncation of the snout, it runs antero-dorsally (as it originates mainly from the	Abnormal, because due to the truncation of the snout, it runs antero-dorsally (as it originates mainly from the	Seemingly missing	Seemingly missing

				region of the facial tuber, which is markedly more posterior than normally) to reach the eye region, and not posterodorsally as normally; that is, basically the size and direction of the fibers changes, but the attachments are mainly normal	region of the facial tuber, which is markedly more posterior than normally) to reach the eye region, and not posterodorsally as normally; that is, basically the size and direction of the fibers changes, but the attachments are mainly normal	region of the facial tuber, which is markedly more posterior than normally) to reach the eye region, and not posterodorsally as normally; that is, basically the size and direction of the fibers changes, but the attachments are mainly normal	the superior part of buccinator area, goes diagonally forward and inserts directly in front of the orbita. The other part lies more superficial, originate from the posterior area of the cheek from the sphincter colli profundus, and inserts onto the orbicularis oculi in the area directly under the orbita	region of the facial tuber, which is markedly more posterior than normally) to reach the eye region, and not posterodorsally as normally; that is, basically the size and direction of the fibers changes, but the attachments are mainly normal	the facial tuber, which is markedly more posterior than normally) to reach the eye region, and not posterodorsally as normally; that is, basically the size and direction of the fibers changes, but the attachments are mainly normal	region of the facial tuber, which is markedly more posterior than normally) to reach the eye region, and not posterodorsally as normally; that is, basically the size and direction of the fibers changes, but the attachments are mainly normal, although on the left side of the head the dorsal attachment was more anterior to the eye region (where it normally attaches in normal newborn lambs)	region of the facial tuber, which is markedly more posterior than normally) to reach the eye region, and not posterodorsally as normally; that is, basically the size and direction of the fibers changes, but the attachments are mainly normal	region of the facial tuber, which is markedly more posterior than normally) to reach the eye region, and not posterodorsally as normally; that is, basically the size and direction of the fibers changes, but the attachments are mainly normal		
Sphincter colli profundus pars auricularis (= 'Parotido-auricularis' or 'Auricularis inferior et depressor auriculae' sensu May 1964) (Note: the mandibuloauricular is, often named 'styloauricularis' in ruminants, is absent in goats/sheep)	Deep to platysma cervicale, inserts dorsally to region of the ear	Mainly normal	Mainly normal	Mainly normal	Mainly normal	Mainly normal	Mainly normal	Mainly normal	Mainly normal	Mainly normal	Mainly normal	Mainly normal	Mainly normal	Mainly normal
Zygomaticus	From zygomatic bone to angle of mouth/upper lip	Mainly normal	Mainly normal	Mainly normal	Mainly normal	Mainly normal	Mainly normal	Mainly normal	Mainly normal	Mainly normal	Mainly normal	Mainly normal	Mainly normal	Mainly normal
Levator labii superioris alaeque nasi (= 'Levator nasolabialis' sensu	From skin if frontal and nasal regions, and from orbicularis oculi,	Mainly normal	Mainly normal	Mainly normal	Mainly normal	Mainly normal	Mainly normal	Mainly normal	Mainly normal	Mainly normal	Mainly normal	Mainly normal	Mainly normal	Seemingly missing

May 1964)	meeting its counterpart at the midline, to nostril and upper lip													
Nasalis (= ‘Dilatator naris apicalis’ or ‘Transversus nasi’ sensu May 1964)	From premaxilla (‘incisive bone’) tonostril, meeting its counterpart at a median raphe	Not analyzed	Not analyzed	Not analyzed	Not analyzed	Not analyzed	Not analyzed	Not analyzed	Not analyzed	Not analyzed	Not analyzed	Not analyzed	Not analyzed	Seemingly missing
Dilatator naris lateralis (= ‘Caninus’ sensu Getty 1975, who confused the identity of the true caninus, which he named ‘depressor labii maxillaris’, see below) (Note: the dilatator naris alaris is seemingly poorly developed in the ox (Getty 1975) and very reduced/absent in sheep, as it was not described as a separate muscle by May 1964, nor found by us; according to Getty 1975 in ruminants it is usually fused with the levator anguli oris facialis, levator labii superioris alaeque nasi, levator labii superioris and dilatator naris lateralis, originating from the premaxilla and lateral nasal cartilages, and inserting onto the nostril)	From facial tuber of maxilla to nostril	Wide but short because of truncation of snout	Mainly normal	Mainly normal	Mainly normal	Not analyzed	Not analyzed	On the left side it originated as usual from facial tuber, but on the right side it originated anteriorly to the facial tuber; on both sides it attached mainly to the ‘proboscis’; moreover, it was deeply fused with the levator anguli oris facialis and with the levator labii superioris on the right side, only two of these three muscles being really seen on that right side	On the right side only a muscle was present where the dilatator naris lateralis and levator anguli oris facialis are usually present, while on the left side both muscles were present as distinct structures	Seemingly missing	On the right side only a muscle was present where the dilatator naris lateralis and levator anguli oris facialis are usually present; it was difficult to analyze these muscles on the left side	Seemingly missing, or at least not clearly present as separate muscle/highly modified	On both the left and right there was seemingly only a muscle where normally lie the levator anguli oris facialis and the dilatator naris lateralis	Seemingly missing
Levator labii superioris (= ‘Levator labii	From facial tuber of maxilla toupper lip and	Wide but short because of truncation of	Mainly normal	Wide but short because of truncation of	Mainly normal	Wide but short because of truncation of	Wide but short because of truncation of	On the left side it originated as usual from facial	Wide but short because of truncation of snout	Wide but short because of truncation of	Very peculiar, very well developed,	Seemingly missing, or at least not clearly	Very peculiar, very well developed,	Very peculiar, running from the facial tuber

maxillaris‘ or ‘Levator labii superioris proprius’ sensu May 1964)	skin between nostrils, meeting its counterpart at the midline	snout		snout		snout	snout	tuber, but on the right side it originated anteriorly to the facial tuber; on both sides it attached mainly to the ‘proboscis’; moreover, it was deeply fused with the levator anguli oris facialis and with the dilatator naris lateralis on the right side, only two of these three muscles being really seen on that right side		snout	running from the facial tuber almost completely dorsomedially to form a ‘constrictor’ that met its counterpart at the midline just above the nose, i.e. it did not go at all to the region of the nasal proboscis	present as separate muscle/highly modified	running from the facial tuber almost completely dorsomedially to form a ‘constrictor’ that met its counterpart at the midline just above the nose, i.e. it did not go at all to the region of the nasal proboscis	almost completely dorsomedially to form a ‘constrictor’ that met its counterpart at the midline just above the nose, i.e. it did not go at all to the region of the nasal proboscis
Levator anguli oris facialis (= ‘Depressor labii maxillaris’ or ‘Depressor labii inferioris’ sensu May 1964) (Note: this is clearly the true levator anguli oris facialis because it is the only one of the small muscles in the region that goes to the upper lip, and moreover is the more inferior and deep one, fusing with the buccinatorius)	From maxilla to nostril and upper lib	Mainly normal	Mainly normal	Mainly normal	Mainly normal	Not analyzed	Not analyzed	On the left side it originated as usual from facial tuber, but on the right side it originated anteriorly to the facial tuber; on both sides it attached mainly to the ‘proboscis’; moreover, it was deeply fused with the dilatator naris lateralis and with the levator labii superioris on the right side, only two of these three muscles being really seen on that right side	On the right side only a muscle was present where the dilatator naris lateralis and levator anguli oris facialis are usually present, while on the left side both muscles were present as distinct structures	Seemingly missing	On the right side only a muscle was present where the dilatator naris lateralis and levator anguli oris facialis are usually present; it was difficult to analyze these muscles on the left side	Seemingly missing, or at least not clearly present as separate muscle/highly modified	On both the left and right there was seemingly only a muscle where normally lie the levator anguli oris facialis and the dilatator naris lateralis	Seemingly missing
Depressor labii inferioris (‘Depressor labii mandibularis’ sensu May 1964; note that the depressor anguli oris is not present as a separate, well-	From mandible to inferior lip and adjacent skin	Mainly normal	Mainly normal	Mainly normal	Mainly normal	Mainly normal	Mainly normal	Mainly normal	Mainly normal	Mainly normal	Mainly normal	Mainly normal	Mainly normal	Mainly normal

defined muscle)														
Orbicularis oris (note: the 'incisivus mandibularis/superior' and 'incisivus maxillaris/superior' sensu May 1964 are part of the orbicularis oris sensu the present work)	From superior and inferior lips to mandible and skin around mouth	Mainly normal	Mainly normal	Mainly normal	Mainly normal	Mainly normal	Mainly normal	Mainly normal	Mainly normal	Mainly normal	Mainly normal	Mainly normal	Mainly normal	Mainly normal
Buccinatorius	From the alveolar border of maxilla and mandible to the entire length of the cheek	Mainly normal	Mainly normal	Mainly normal	Mainly normal	Mainly normal	Mainly normal	Mainly normal	Mainly normal	Mainly normal	Mainly normal	Mainly normal	Mainly normal	Mainly normal
Mentalis	From body of mandible near symphysis toskin of chin region	Mainly normal	Mainly normal	Mainly normal	Mainly normal	Mainly normal	Mainly normal	Mainly normal	Mainly normal	Mainly normal	Mainly normal	Mainly normal	Mainly normal	Mainly normal
Orbicularis oculi	From palpebral ligament and lacrimal tubercle toskin of eyelid region	Mainly normal	Mainly normal	Mainly normal	Mainly normal	Mainly normal	Mainly normal	Mainly normal	Mainly normal	Mainly normal	Mainly normal	Mainly normal	Mainly normal	Mainly normal
Frontalis (note that the levator anguli oculi medialis, or 'corrugator supercilii', is not present in ruminants according to Getty 1975, and not in sheep according to May 1964)	From skin of frontal region to eye region and skin of the dorsal surface of forehead	Mainly normal	Mainly normal	Mainly normal	Mainly normal	Mainly normal	Mainly normal	Mainly normal	Mainly normal	Mainly normal	Mainly normal	Mainly normal	Mainly normal	Mainly normal
Zygomaticoauricularis (= 'Zygomatico-auricularis' sensu May 1964) (Note: we are not including in this study the numerous small facial muscles related to the movements of the ear, but our	From zygomatic arch to auricular cartilage	Mainly normal	Mainly normal	Mainly normal	Mainly normal	Mainly normal	Mainly normal	Mainly normal	Mainly normal	Mainly normal	Mainly normal	Mainly normal	Mainly normal	Mainly normal

Zygomaticoauricularis (= 'Zygomatico-auricularis' sensu May 1964) (Note: we are not including in this study the numerous small facial muscles related to the movements of the ear, but our dissections indicate that their presence, and attachments, were constant and similar to the normal phenotype in all abnormal specimens we analyzed, both the more and the less defective ones)	From zygomatic arch to auricular cartilage	Mainly normal	Mainly normal	Mainly normal	Mainly normal	Mainly normal	Mainly normal	Mainly normal	Mainly normal	Mainly normal	Mainly normal	Mainly normal	Mainly normal	Mainly normal
Levator palpebrae superioris	From pterygoid crest to palpebral fascia of superior eyelid	Mainly normal	Mainly normal	Mainly normal	Mainly normal	Mainly normal	Mainly normal	Mainly normal	Mainly normal	Mainly normal	Mainly normal	Mainly normal	Not analyzed	Mainly normal
Rectus superioris	From sphenoid bone to eye	Mainly normal	Mainly normal	Mainly normal	Mainly normal	Mainly normal	Mainly normal	Mainly normal	Mainly normal	Mainly normal	Mainly normal	Mainly normal	Not analyzed	Mainly normal
Rectus inferioris	From sphenoid bone to eye	Abnormal, fused with rectus medialis, although its attachments are as normal	Mainly normal	Mainly normal	Mainly normal	Not analyzed	Mainly normal	Mainly normal	Mainly normal	Inferior rectus seemed to be a wide muscle extending all the way to the midline and thus probably meeting with the counterpart at the midline, being possibly a single muscle for the two eyes as a whole	Inferior rectus seemed to be a wide muscle extending all the way to the midline and thus probably meeting with the counterpart at the midline, being possibly a single muscle for the two eyes as a whole	Mainly normal	Not analyzed	Mainly normal
Rectus lateralis	From sphenoid bone to eye	Mainly normal	Mainly normal	Mainly normal	Mainly normal	Not analyzed	Mainly normal	Mainly normal	Mainly normal	Mainly normal	Mainly normal	Mainly normal	Not analyzed	Mainly normal
Rectus medialis	From region of optic foramen to eye	Abnormal, fused with rectus inferioris, although its attachments are	Mainly normal	Mainly normal	Mainly normal	Not analyzed	Mainly normal	Mainly normal	Mainly normal	Mainly normal	Seemingly missing	Seemingly missing	Not analyzed	Missing

**Muscles of the ear**														
Zygomaticoauricularis (= 'Zygomatico-auricularis' sensu May 1964) (Note: we are not including in this study the numerous small facial muscles related to the movements of the ear, but our dissections indicate that their presence, and attachments, were constant and similar to the normal phenotype in all abnormal specimens we analyzed, both the more and the less defective ones)	From zygomatic arch to auricular cartilage	Mainly normal	Mainly normal	Mainly normal	Mainly normal	Mainly normal	Mainly normal	Mainly normal	Mainly normal	Mainly normal	Mainly normal	Mainly normal	Mainly normal	Mainly normal
Masseter	Superficial layer from facial tuber, maxilla and zygomatic arch portion of zygomatic bone; middle and deep layers from zygomatic arch, malar bone, and maxilla; superficial layer to caudal and ventral parts and to angle of mandible; middle layer to caudal and ventral borders and angle of ramus of mandible; deep layer to neck of ramus and articular surface of mandible, coronoid process,	Mainly normal	Mainly normal	Mainly normal	Mainly normal	Mainly normal	Mainly normal	Mainly normal	Mainly normal	Mainly normal	Mainly normal	Mainly normal	Mainly normal	Mainly normal

	and rostral part of ramus of mandible													
Temporalis	From termporal bone, frontal and temporal crests and zygomatic arch to coronoid process of mandible	Mainly normal	Mainly normal	Mainly normal	Mainly normal	Mainly normal	Mainly normal	Mainly normal	Mainly normal	Mainly normal	Mainly normal	Mainly normal	Mainly normal	Mainly normal
Pterygoideus medialis	From palatine bone, pterygoid bone and pterygoid process of basisphenoid bone, to medial surface of mandible	Mainly normal	Mainly normal	Mainly normal	Mainly normal	Mainly normal	Mainly normal	Mainly normal	Mainly normal	Mainly normal	Mainly normal	Mainly normal	Mainly normal	Mainly normal
Pterygoideus lateralis	Only one head; from pterygpoalatine fossa and pterygoid process of basisphenoid to medial surface of mandible and base of coronoid process	Mainly normal	Mainly normal	Mainly normal	Mainly normal	Mainly normal	Mainly normal	Mainly normal	Mainly normal	Mainly normal	Mainly normal	Mainly normal	Mainly normal	Mainly normal
Digastricus	It is crossed by the stylohyoideus, and the anterior and posterior digastric bellies are connected by thin intermediate tendon, which does not perforate the stylohyoideus; the anterior head of digastricus is deeply blended with mylohyoideus; digastricus as a whole runs from jugular ('paramastoid' sensu May 1964) process of	Mainly normal	Mainly normal	Mainly normal	Mainly normal	Mainly normal	Mainly normal	Mainly normal	Mainly normal	Mainly normal	Mainly normal	Mainly normal	Mainly normal	Mainly normal

	occipital bone to mandible													
Mylohyoideus	Has superficial and deep bundles in sheep according to May 1964; both bundles run from mylohyoid line of mandible to hyoid bone and to midline raphe	Mainly normal	Mainly normal	Mainly normal	Mainly normal	Mainly normal	Mainly normal	Mainly normal	Mainly normal	Mainly normal	Mainly normal	Mainly normal	Mainly normal	Mainly normal
Stylohyoideus	Has a long thin tendon of origin; from muscular angle of stylohyoid bone ('great cornu of hyoid bone' sensu May 1964) tobasihyoid bone ('body of hyoid bone' sensu May 1964)	Much shorter than normal, but basically same attachments	Mainly normal	Much shorter than normal, but basically same attachments	Much shorter than normal, but basically same attachments	Much shorter than normal, but basically same attachments	Much shorter than normal, but basically same attachments	Much shorter than normal, but basically same attachments	Much shorter than normal, but basically same attachments	Much shorter than normal, but basically same attachments	Much shorter than normal, but basically same attachments, and deeply blended with the posterior belly of the digastricus	Much shorter than normal, but basically same attachments	Much shorter than normal, but basically same attachments	Much shorter than normal, but basically same attachments
Jugulohyoideus (= 'Occipitohyoideus' sensu May 1964*)*	From jugular process of occipital bone tostylohyoid bone	Mainly normal	Mainly normal	Mainly normal	Mainly normal	Mainly normal	Mainly normal	Mainly normal	Mainly normal	Mainly normal	Mainly normal	Mainly normal	Mainly normal	Mainly normal
Geniohyoideus	Contacts with counterpart at midline; from mandible just near to symphysis, to basihyoid	Mainly normal	Mainly normal	Mainly normal	Mainly normal	Mainly normal	Mainly normal	Mainly normal	Mainly normal	Mainly normal	Mainly normal	Mainly normal	Mainly normal	Mainly normal
Ceratohyoideus	From ceratohyoid (and also stylohyoid according to May 1964) tothyrohyoid	Mainly normal	Mainly normal	Mainly normal	Mainly normal	Mainly normal	Mainly normal	Mainly normal	Mainly normal	Mainly normal	Mainly normal	Mainly normal	Mainly normal	Mainly normal
Hyoideus transversus	Connects ceratohyoid cartilages	Mainly normal	Mainly normal	Mainly normal	Mainly normal	Mainly normal	Mainly normal	Mainly normal	Mainly normal	Mainly normal	Mainly normal	Mainly normal	Mainly normal	Mainly normal
Styloglossus	From stylohyoid to tongue	Mainly normal	Mainly normal	Mainly normal	Mainly normal	Mainly normal	Mainly normal	Mainly normal	Mainly normal	Mainly normal	Mainly normal	Mainly normal	Mainly normal	Mainly normal
Hyoglossus	From basihyoid and lingual process of	Mainly normal	Mainly normal	Mainly normal	Mainly normal	Mainly normal	Mainly normal	Mainly normal	Mainly normal	Mainly normal	Mainly normal	Mainly normal	Mainly normal	Mainly normal

Hyoglossus	From basihyoid and lingual process of thyrohyoid (but from stylohyoid according to May 1964, what seems to be a mistake) to tongue	Mainly normal	Mainly normal	Mainly normal	Mainly normal	Mainly normal	Mainly normal	Mainly normal	Mainly normal	Mainly normal	Mainly normal	Mainly normal	Mainly normal	Mainly normal
Genioglossus	From mandible (chin) just near to symphysis, to tongue	Mainly normal	Mainly normal	Mainly normal	Mainly normal	Mainly normal	Mainly normal	Mainly normal	Mainly normal	Mainly normal	Mainly normal	Mainly normal	Mainly normal	Mainly normal
Hyoepiglotticus	Contacts its counterpart at midline; innervated by hypoglossal nerve, so seems to be hypobrachial muscle, not true laryngeal or pharyngeal muscle; runs from basihyoid to epiglottic cartilage	Not analyzed	Not analyzed	Not analyzed	Not analyzed	Not analyzed	Not analyzed	Not analyzed	Not analyzed	Not analyzed	Not analyzed	Not analyzed	Not analyzed	Not analyzed
Cricothyroideus	From posterior border and lateral surface of arch of cricoid cartilage tolateral surface of lamina of, and lateral surface of, thyroid cartilage	Mainly normal	Mainly normal	Mainly normal	Mainly normal	Mainly normal	Mainly normal	Mainly normal	Mainly normal	Mainly normal	Mainly normal	Mainly normal	Mainly normal	Mainly normal
Cricoarytenoideus posterior (= 'Cricoarytenoideus dorsalis')	From dorsal surface and median ridge of lamina of cricoid cartilage toarytenoid cartilage	Mainly normal	Mainly normal	Mainly normal	Mainly normal	Mainly normal	Mainly normal	Mainly normal	Mainly normal	Mainly normal	Mainly normal	Mainly normal	Mainly normal	Mainly normal
Cricoarytenoideus lateralis	From rostral border and lateral surface of arch of cricoid cartilage to muscular	Mainly normal	Mainly normal	Mainly normal	Mainly normal	Mainly normal	Mainly normal	Mainly normal	Mainly normal	Mainly normal	Mainly normal	Mainly normal	Mainly normal	Mainly normal

Arytenoideus (= 'Arytenoideus transversus')	Unpaired, from arytenoid cartilages to arytenoid cartilages	Mainly normal	Mainly normal	Mainly normal	Mainly normal	Mainly normal	Mainly normal	Mainly normal	Mainly normal	Mainly normal	Mainly normal	Mainly normal	Mainly normal	Mainly normal
Thyroarytenoideus	From thyroid cartilage to arytenoid cartilage	Mainly normal	Mainly normal	Mainly normal	Mainly normal	Mainly normal	Mainly normal	Mainly normal	Mainly normal	Mainly normal	Mainly normal	Mainly normal	Mainly normal	Mainly normal

Musculoskeletal study of cebocephalic and cyclopic lamb heads illuminates links between normal and abnormal development, evolution and human pathologies

Authors: Rui Diogo, Daria Razmadze, Natalia Siomava, Nora Douglas, Jose S. M. Fuentes, Andre Duerinckx

Supplementary Information 2, including summarized methods, as well as results, of morphometric analysis

For the morphometric analysis of all the left and right sides of the skull of each abnormal specimen dissected by us, we first removed all the soft tissues of those specimens, and then used landmarks that are consistently used for morphometric analysis of mammalian skulls (junction of the front tooth and maxilar bone; connection between the zygomatic and temporal bones in the zygomatic arch; facil tuber; around the eye; see e.g. Bookstein 1991), in order to run our analyses, which included Proctustes ANOVA. Skull halves were photographed on a flat horizontal surface with a camera Nikon D5000 fixed in a strict vertical position. Shape of skulls was analyzed using landmark-based geometric morphometric methods (Bookstein, 1991; Rohlf, 1990). Images were digitized using tpsUtil (Rohlf, 2004) and tpsDig2 (Rohlf, 2010). Series of landmarks along the mandible and eye hole were placed equidistant along the curvature. We applied the generalized Procrustes analysis (GPA) (Dryden and Mardia, 2002; Slice, 2005) in MorphoJ 1.05f (Klingenberg, 2008, 2011) to align the landmarks. Right and left halves were analyzed separately. Shape variation was studied by performing (1) Principal Component Analysis (PCA) of the data with the allometric component included (total shape variation) and (2) PCA after the regression of the centroid size on shape (pure shape) and visualizing the shape with a scatter plot and morphological differences with thin-plate spline (TPS) deformation grids (Bookstein, 1991; James Rohlf and Marcus, 1993; Slice, 2005; Thompson, 1917). To visualize the association between size and shape, we plotted shape scores against WCS. The amount of shape variation was given as a percentage of the total variation around the sample mean. The percentage numbers were computed to shows the relative importance of allometry for shape variation in each part of the skull. A permutation test with 10,000 runs (Good, 1994; Pitman, 1937) was applied to test independence between size and shape changes. A discriminant function analysis (DFA) and canonical variate analysis (CVA) were used to distinguish between groups. Procrustes ANOVA was used to estimate the fluctuating asymmetry (FA, asymmetric variation within one individual) and directional asymmetry (DA, one side is systemically different from the other one) in both centroid size and shape.

Because the lower jaw is movable, it is difficult to always keep it exactly within the same position in the skulls: because this factor may affect the overall result we analyzed the jaw separately. The results for the lower jaw are shown in Fig. 6 of the main paper, and are also shown in the figure just below. Other parts of the skull were also have to split into regions. For example, when we analyze the snout, we cannot use true cyclops (stage 4) because they basically don't have a ossified snout. On the other hand, we can analyze the brain case in all specimens. Thus, we obtained different groupings depending on what regions were included in the various morphometric analyses. The main results are summarized below:

Lower jaw:

Fig. PCA Lower jaw. Variation of shape of mandible in animals with different degree of defects. PCA of shape scatter plot (PC1 and PC2) and associated shape changes of non-allometric shape component of eye curvature and snout. The TPS deformation grids illustrate shape changes indicating the relative shifts of landmarks along the PC1 axes with PC scale factor +/-0.1 and along the PC2 axes with PC scale factor +/-0.05. Shape of lower jaw is significantly different between degrees 2, 3 and 4. The difference between groups is clear along PC1, which is mainly a change in curvature of lower surface of mandible and position of coronoid and condylar processes relative to body of the mandible.

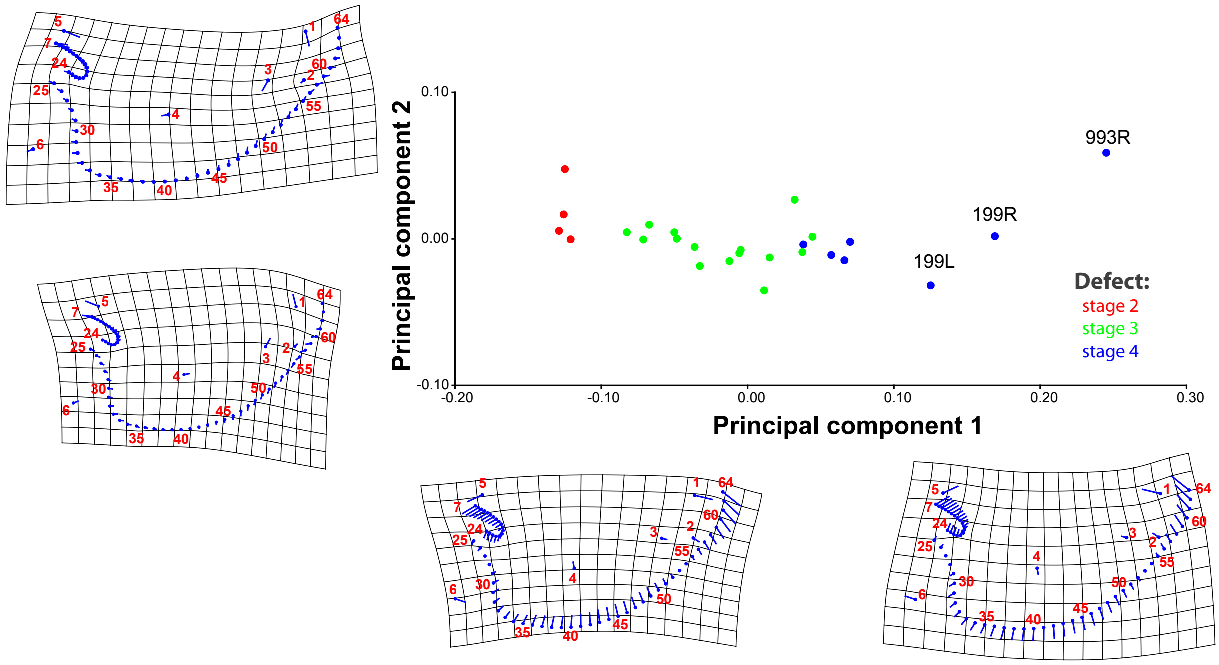

- Contrarily to what we found concerning soft tissue pattern (i.e. usual asymmetry between left and right sides), there is no directional asymmetry (DA, i.e. in which one side would be systemically different from the other one) in the skull in size (Centroid Size, p= 0.7863) or shape (p= 0.7141) revealed by Proctustes ANOVA (see Table Proctustes ANOVA below):

Classifiers used for the Procrustes ANOVA:
Individuals: Specimen
Sides: Side
Error 1: Repeat

Centroid size:

Effect	SS	MS	df	F	P (param.)
Individual	15094186.659813	1509418.665981	10	8.32	0.0012
Side	14060.301372	14060.301372	1	0.08	0.7863
Ind * Side	1813595.719212	181359.571921	10	1602.90	<.0001
Error 1	2489.184785	113.144763	22		

Shape, Procrustes ANOVA:

Effect	SS	MS	df	F	P (param.)	Pillai tr.	P (param.)
Individual	0.25472545	0.0002054237	1240	10.78	<.0001		
Side	0.00217879	0.0000175709	124	0.92	0.7141		
Ind * Side	0.02362867	0.0000190554	1240	48.85	<.0001		
Error 1	0.00106409	0.0000003901	2728				

- However, there is a strong fluctuating asymmetry (FA, asymmetric variation within one individual) in both size (Centroid Size, p<0.005) and shape (p<0.0001) (Table Proctustes ANOVA; AL PCA, specimen 199). FA [illegible]

and right sides. The left and right sides develop as more or less separate copies of each other within the same genome and in *nearly* the same environment. There are a variety of random processes at the molecular and cellular levels that can affect development and therefore may produce small differences between body parts even if genetic and environmental differences are absent. The differences between left and right sides are an opportunity to measure this variation and FA can therefore be used as a measure of the 'imprecision' of developmental processes, or *developmental instability*.

- All analysis ware performed with true landmarks (LM), no semilandmark (semiLM) as the curvature of the lower jaw is very different between individuals. Relaxation of semiLM leads to their rearrangement and does not depict the trend. Repeated measurements show that the error of LM analysis is relatively low (Table Proctustes ANOVA) and suggest that the results obtained are reliable.
- R and L sides were not averages as each of them includes important information information.
- When the allometric component were included, the Principal Component 1 (PC1) explains 84.5% of the shape variation. Other PCs explain <5% (PC2 4.2%). Transformation grids located in corners of the graph illustrate shape changes along PC1 and PC2. For the PC1, it's mainly changing of the jaw curvature. In more defective specimens, the lower jaw is wider and bent upward; in less defective specimens, the jaw is straighter. Another shape alteration is the widening of the condylar process in cyclop specimens (stage 4).
- The allometric component is very strong and can explain 49.1% of the variation (p<0.0001). In general, most defective specimens are smaller than others. However, stages 3 and 4 significantly overlap in size, as can see in this figure showing Regression:

Fig. Regressions. Regression of centroid size on shape. A distribution of different shapes of mandible (top panel), brain case (middle panel), and face (bottom panel) across specimens with different skull sizes. There is a clear distribution of mandible shape along size of skull and degree of defect. Specimens with stage 4 have the largest and the smallest brain cases. Animals with the stage 2 tend to have larger frontal parts of the skull than animals with stage 3, but they still substantially overlap.

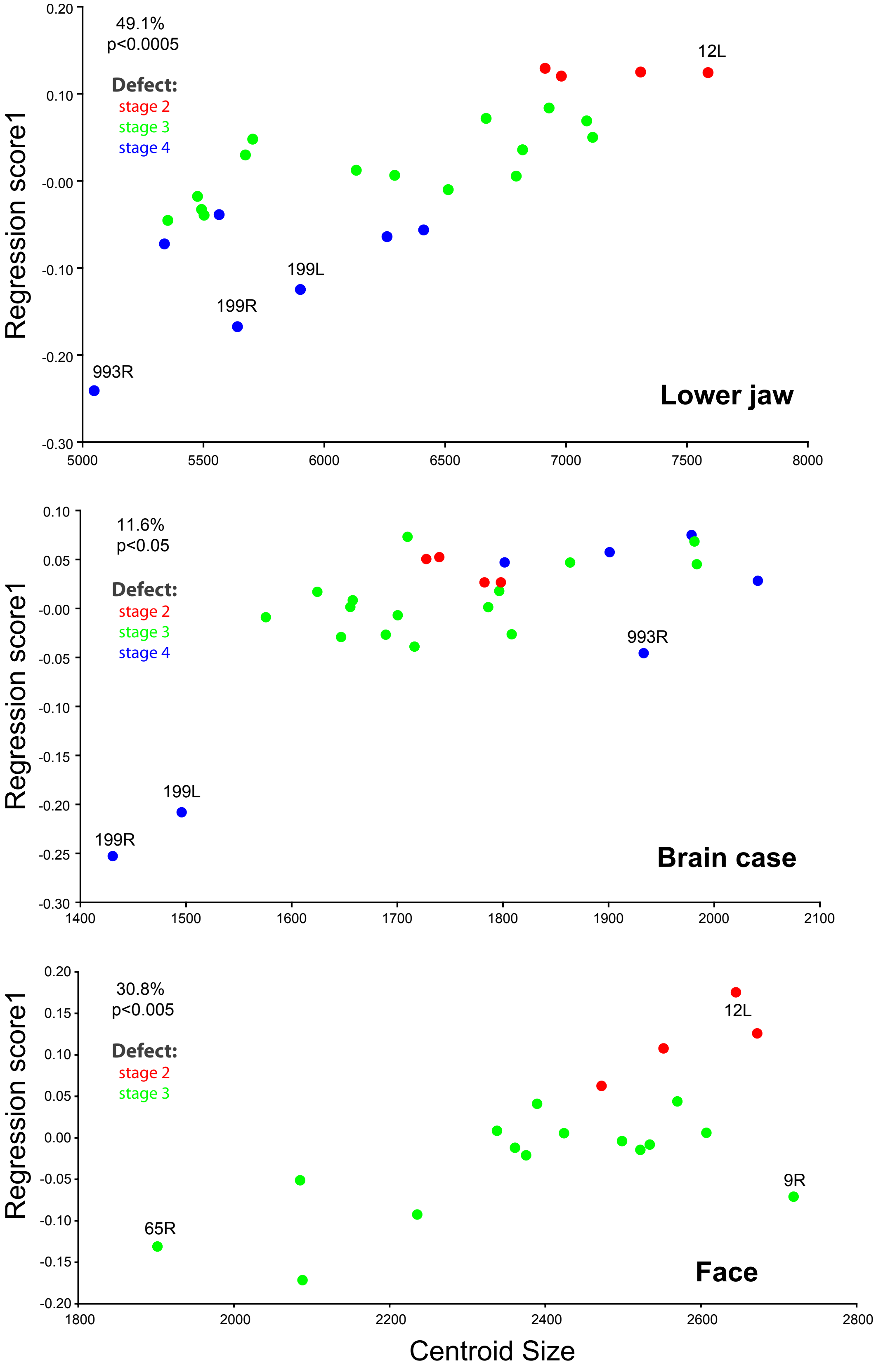

- Correction for size removes the difference between stages 2 and 3 (Discriminant Function Analysis, DFA and Canonical Variate Analysis, CVA) but shape changes along PC1 and PC2 remain similar (not shown). Stage 4 is always statistically different (p<0.05) from the other two (Table CVA, top: allometric component included, bottom: excluded).

```
Procrustes distances among groups:

        2       3
3          0.1115
4          0.2369          0.1288

P-values from permutation tests (10000 permutation rounds) for Procrustes distances among groups:

        2       3
3       0.0001
4       0.0018  <.0001
```

```
Procrustes distances among groups:

        2       3
3          0.0329
4          0.0986          0.0816

P-values from permutation tests (10000 permutation rounds) for Procrustes distances among groups:

        2       3
3       0.2777
4       0.0287  0.0024
```

- Specimens 199 and 993 (the most defective, stage 4, specimens) are often outliers in the morphometric analyses. Specimen 993 has the jaw most bent upward. Specimen 199 has almost no brain, but the analysis shows that not only the brain case is defective but also the facial bones and the lower jaw, in particular.

Table Proctustes ANOVA	PC1 (Al)	PC2(Al)	PC3 (Al)	Predicted Allometry	PC1 (Shape)	PC2 (Shape)	PC3 (Shape)
Lower Jaw (All)	84.462	4.225		49.0954% (p<.0001)	72.120	8.241	
Back Skull (All)	46.178	16.432	12.109	11.7204% (p=0.0202)	48.077	16.915	10.050
St. 4: Front Skull	80.475	12.125	4.032	36.3108% (p=0.1078)			
St. 2-3: Front Skull	66.318	11.203	7.166	30.8352% (p=0.0016)	55.253	16.189	9.650

PCA Brain case:

Fig. PCA Brain case. Variation of shape of brain case in animals with different degree of defects. PCA of shape scatter plot (PC1 and PC2) and associated shape change of non-allometric shape component of skulls. The TPS deformation grids illustrate shape changes indicating relative shifts of landmarks along axes with the PC scale factor +/-0.1. Specimens 993 and 199 are outliers. Other specimens do not show essential overlap in the shape of the brain case.

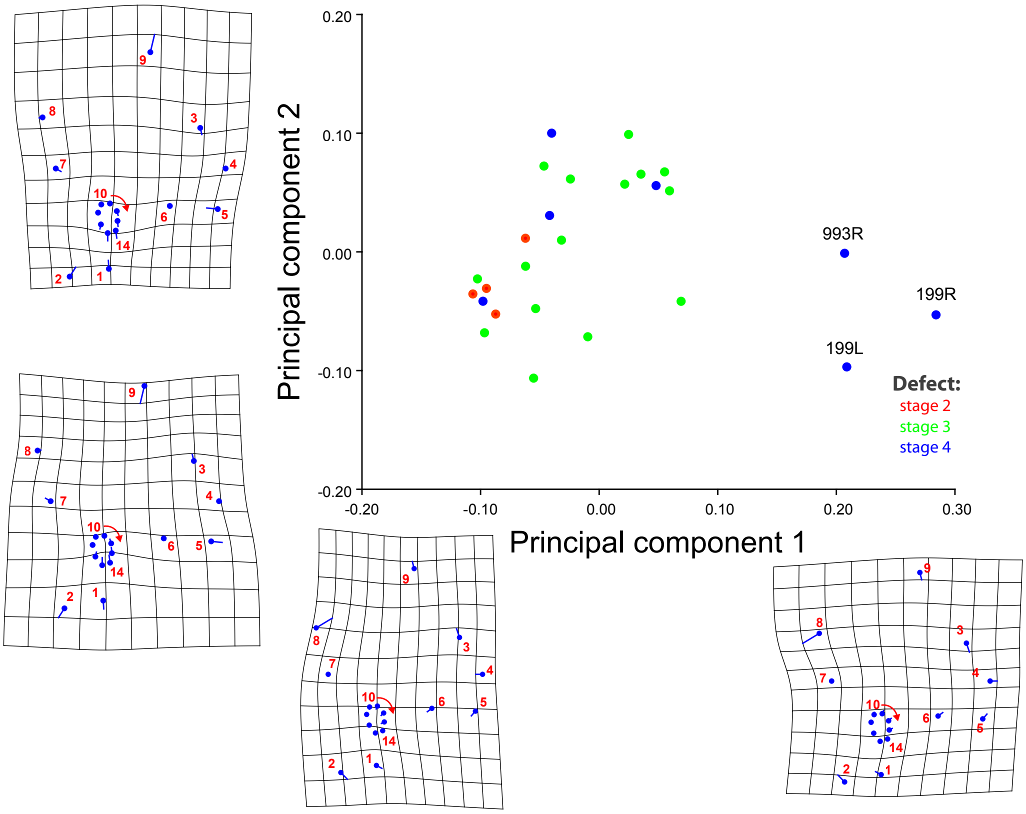

PCA Face Stages 2 and 3:

Fig. PCA Face (Stages 2 and 3). Variation of shape of frontal part of skull in animals with different degree of defects. PCA of shape scatter plot (PC1 and PC2) and associated shape changes of non-allometric shape component of eye and snout. The TPS deformation grids illustrate shape changes indicating the relative shifts of landmarks along the axes with PC scale factor +/-0.15. Shape of frontal part of skull is significantly different between stages 2 and 3. The difference between groups is present along PC1, which mainly concerns the length of the snout.

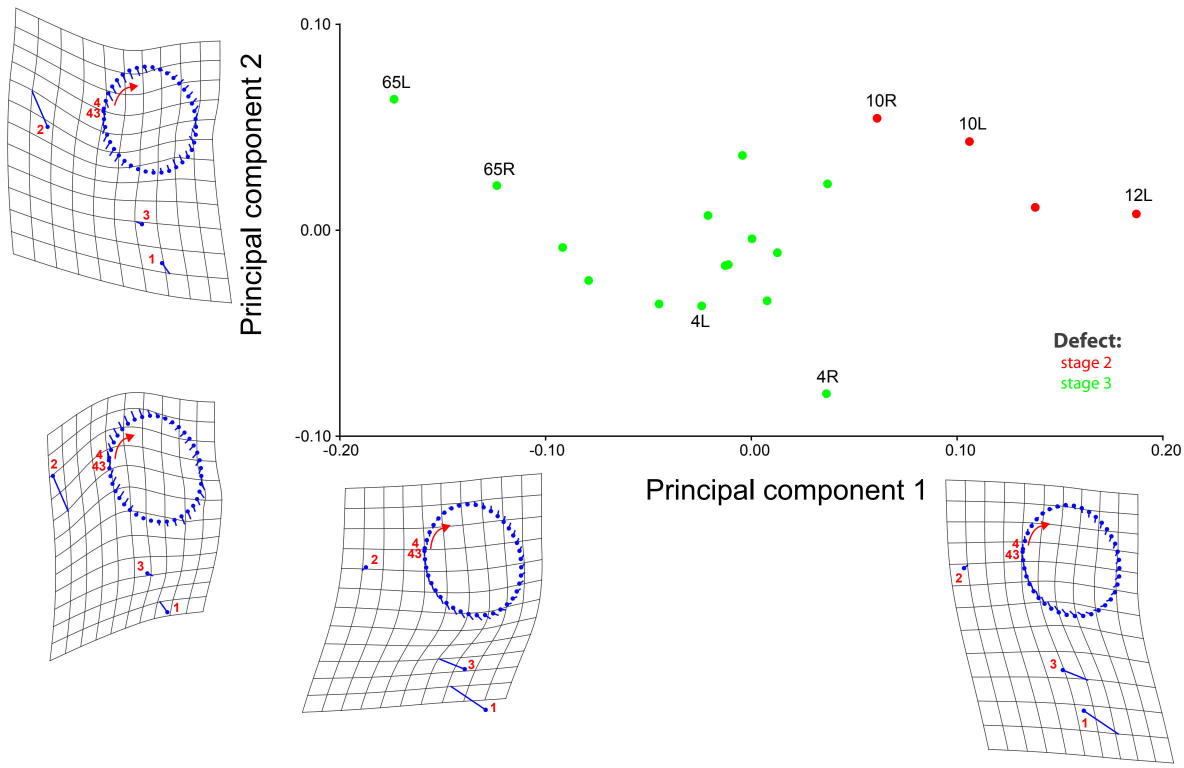

PCA Face Stage 4:

Fig. PCA Face (Stage 4). Variation of shape of frontal part of skull in animals with stage 4. PCA of shape scatter plot (PC1 and PC2) and associated shape changes of non-allometric shape component of the eye curvature and snout. The TPS deformation grids illustrate shape changes indicating relative shifts of landmarks along axes with PC scale factor +/-0.1. Specimens 3 and 11 have similar shapes of the face; specimen 993 is rather unique as it is distant from other specimens along both PC1 and PC2.

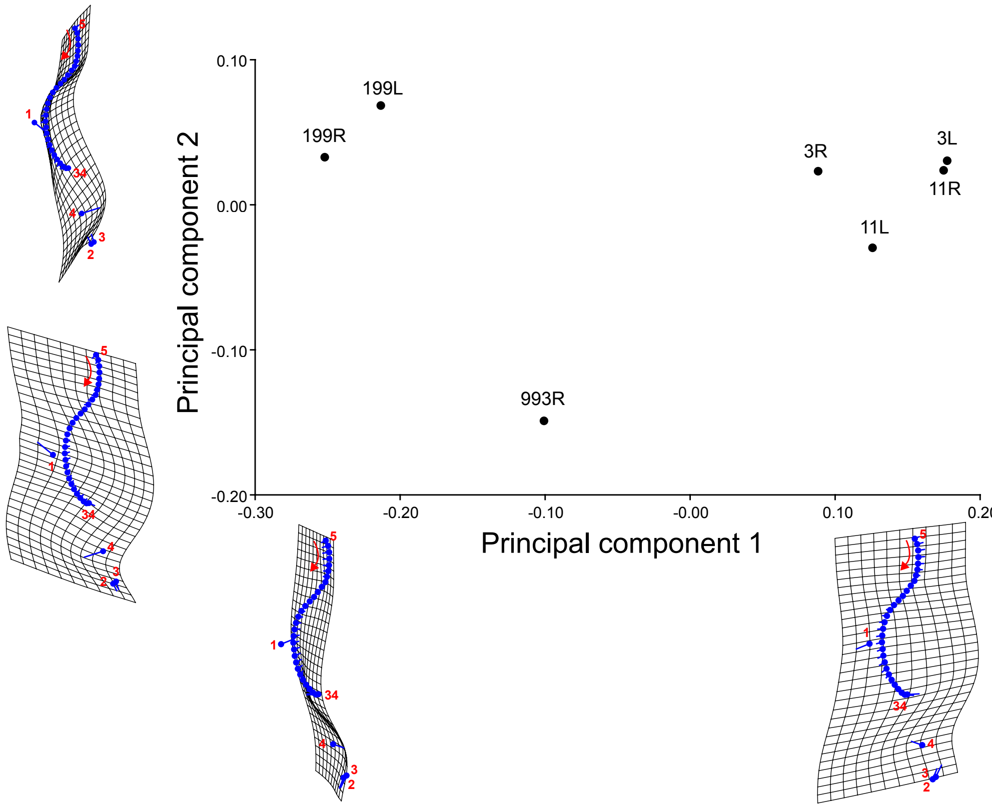

CVA: Lower Jaw

Procrustes distances among groups:

	2	3
3	0.1113	
4	0.2366	0.1289

P-values from permutation tests (10000 permutation rounds) for Procrustes distances among groups:

	2	3
3	0.0001	
4	0.0036	<.0001

Procrustes distances among groups:

	2	3
3	0.0340	
4	0.0994	0.0820

P-values from permutation tests (10000 permutation rounds) for Procrustes distances among groups:

	2	3
3	0.2526	
4	0.0272	0.0026

CVA: Back Skull

Procrustes distances among groups:

	2	3
3	0.0928	
4	0.1784	0.1064

P-values from permutation tests (10000 permutation rounds) for Procrustes distances among groups:

	2	3
3	0.0608	
4	0.0430	0.0432

Procrustes distances among groups:

	2	3
3	0.0912	
4	0.1842	0.1158

P-values from permutation tests (10000 permutation rounds) for Procrustes distances among groups:

	2	3
3	0.0692	
4	0.0172	0.0123

DFA: Non-Cyclops: Front Skull

```
Discriminant Function Analysis 'Discriminant function Non-Cyclops: Front Skull'
Comparison: 2 -- 3

Difference between means:
Procrustes distance:  0.16134242
Mahalanobis distance:      7.8456
T-square:  194.3789,   P-value (parametric): 0.7606
P-values for permutation tests (1000 permutation runs):
Procrustes distance: <.0001
T-square: 0.0170
(Note: The permutation test using the T-square statistic is equivalent to a test using Mahalanobis distance.)

Classification/misclassification tables
Group 1: 2
Group 2: 3
From discriminant function:
True               Allocated to
Group        Group 1        Group 2         Total
Group 1          4              0             4
Group 2          0             15            15
From cross-validation:
True               Allocated to
Group        Group 1        Group 2         Total
Group 1          3              1             4
Group 2          2             13            15
```

```
Discriminant Function Analysis 'Discriminant function ...'
Comparison: 2 -- 3

Difference between means:
Procrustes distance:  0.11082048
Mahalanobis distance:      5.2752
T-square:   87.8768,   P-value (parametric): 0.9125
P-values for permutation tests (1000 permutation runs):
Procrustes distance: <.0001
T-square: 0.9240
(Note: The permutation test using the T-square statistic is equivalent to a test using Mahalanobis distance.)

Classification/misclassification tables
Group 1: 2
Group 2: 3
From discriminant function:
True               Allocated to
Group        Group 1        Group 2         Total
Group 1          4              0             4
Group 2          0             15            15
From cross-validation:
True               Allocated to
Group        Group 1        Group 2         Total
Group 1          2              2             4
Group 2          2             13            15
```

Procrustes ANOVA: Lower Jaw

Asymmetries are computed and displayed as 'R' minus 'L'.

Classifiers used for the Procrustes ANOVA:
Individuals: Specimen
Sides: Side
Error 1: Repeat

Centroid size:

Effect	SS	MS	df	F	P (param.)
Individual	15094186.659813	1509418.665981	10	24.97	<.0001
Side	14060.301372	14060.301372	1	0.23	0.6400
Ind * Side	604531.906404	60453.190640	10	534.30	<.0001
Error 1	2489.184785	113.144763	22		

Shape, Procrustes ANOVA:

Effect	SS	MS	df	F	P (param.)	Pillai tr.	P (param.)
Individual	0.25472545	0.0002054237	1240	10.78	<.0001		
Side	0.00217879	0.0000175709	124	0.92	0.7141		
Ind * Side	0.02362867	0.0000190554	1240	48.85	<.0001		
Error 1	0.00106409	0.0000003901	2728				

Procrustes ANOVA: Back Skull

Procrustes ANOVA: Procrustes ANOVA Back Skull
Dataset: Back Skull

Asymmetries are computed and displayed as 'R' minus 'L'.

Classifiers used for the Procrustes ANOVA:
Individuals: Species
Sides: Side
Error 1: Repeat

Centroid size:

Effect	SS	MS	df	F	P (param.)
Individual	974126.722476	97412.672248	10	5.81	0.0051
Side	48.712114	48.712114	1	0.00	0.9581
Ind * Side	167547.448755	16754.744876	10	884.50	<.0001
Error 1	416.736312	18.942560	22		

Shape, Procrustes ANOVA:

Effect	SS	MS	df	F	P (param.)	Pillai tr.	P (param.)
Individual	0.68393654	0.0022797885	300	4.02	<.0001		
Side	0.03244470	0.0010814899	30	1.91	0.0038		
Ind * Side	0.17017484	0.0005672495	300	24.57	<.0001		
Error 1	0.01523520	0.0000230836	660				

Procrustes ANOVA: Cyclop: Front Skull

Procrustes ANOVA: Procrustes ANOVA Cyclop: Front Skull
Dataset: Cyclop: Front Skull

Asymmetries are computed and displayed as 'R' minus 'L'.

Classifiers used for the Procrustes ANOVA:
Individuals: Species
Sides: Side
Error 1: Repeat

Centroid size:

Effect	SS	MS	df	F	P (param.)
Individual	332959.560670	166479.780335	2	0.77	0.5645
Side	3554.464306	3554.464306	1	0.02	0.9096
Ind * Side	431504.559046	215752.279523	2	3445.69	<.0001
Error 1	375.691112	62.615185	6		

Shape, Procrustes ANOVA:

Effect	SS	MS	df	F	P (param.)	Pillai tr.	P (param.)
Individual	0.38972045	0.0030446910	128	12.02	<.0001		
Side	0.01058280	0.0001653562	64	0.65	0.9704		
Ind * Side	0.03240944	0.0002531987	128	3.00	<.0001		
Error 1	0.03241908	0.0000844247	384				

Procrustes ANOVA: Non-Cyclop: Front Skull

Procrustes ANOVA: Procrustes ANOVA Non-Cyclop: Front Skull
Dataset: Non-Cyclops: Front Skull

Asymmetries are computed and displayed as 'R' minus 'L'.

Classifiers used for the Procrustes ANOVA:
Individuals: Species
Sides: Side
Error 1: Repeat

Centroid size:

Effect	SS	MS	df	F	P (param.)
Individual	1129775.122071	161396.446010	7	4.58	0.0312
Side	775.704994	775.704994	1	0.02	0.8862
Ind * Side	246552.995264	35221.856466	7	560.73	<.0001
Error 1	1005.027915	62.814245	16		

Shape, Procrustes ANOVA:

Effect	SS	MS	df	F	P (param.)	Pillai tr.	P (param.)
Individual	0.20305425	0.0003537530	574	3.64	<.0001		
Side	0.00440171	0.0000536794	82	0.55	0.9994		
Ind * Side	0.05578871	0.0000971929	574	42.89	<.0001		
Error 1	0.00297283	0.0000022659	1312				

References cited in SI2

Bookstein, F.L., 1991. Morphometric tools for landmark data: geometry and biology, Reprint. ed. Cambridge Univ. Press, Cambridge.

Dryden, I.L., Mardia, K.V., 2002. Statistical shape analysis, Reprinted. ed, Wiley series in probability and statistics. Wiley, Chichester.

Good, P., 1994. Permutation Tests: a Practical Guide to Resampling Methods for Testing Hypotheses. Springer New York, New York, NY.

James Rohlf, F., Marcus, L.F., 1993. A revolution morphometrics. Trends Ecol. Evol. 8, 129–132. https://doi.org/10.1016/0169-5347(93)90024-J

Klingenberg, C., 2008. MorphoJ Software/Documentation. Version 1.00i.

Klingenberg, C.P., 2011. MorphoJ: an integrated software package for geometric morphometrics. Mol. Ecol. Resour. 11, 353–357. https://doi.org/10.1111/j.1755-0998.2010.02924.x

Pitman, E.J.G., 1937. Significance Tests Which May be Applied to Samples from any Populations. II. The Correlation Coefficient Test. Suppl. J. R. Stat. Soc. 4, 225. https://doi.org/10.2307/2983647

Rohlf, F.J., 2010. tpsDig, Digitize Landmarks and Outlines, Version 2.17.

Rohlf, F.J., 2004. tpsUtil, File Utility Program, Version 1.54.

Rohlf, F.J., 1990. Morphometrics. Annu. Rev. Ecol. Syst. 21, 299–316. https://doi.org/10.1146/annurev.es.21.110190.001503

Slice, D.E., 2005. Modern Morphometrics, in: Slice, D.E. (Ed.), Modern Morphometrics in Physical Anthropology. Kluwer Academic Publishers-Plenum Publishers, New York, pp. 1–45. https://doi.org/10.1007/0-387-27614-9_1

Thompson, D.W., 1917. On growth and form. Cambridge University Press.
